# Variability in the anthelmintic efficacy of levamisole against gastrointestinal nematodes of cattle, sheep and goats in South Darfur, Sudan

**DOI:** 10.1186/s12917-026-05320-2

**Published:** 2026-02-11

**Authors:** Khalid M. Mohammedsalih, Abdoelnaim I. Y. Ibrahim, Fathel-Rahman Juma, Abdalhakaim A. H. Abdalmalaik, Ahmed Bashar, Georg von Samson-Himmelstjerna, Jürgen Krücken

**Affiliations:** 1 Institute for Parasitology and Tropical Veterinary Medicine, Freie Universität Berlin, Robert-Von-Ostertag-Str. 7, 14163 Berlin, Germany; 2 Veterinary Centre for Resistance Research, Freie Universität Berlin, 14163 Berlin, Germany; 3 Central Research Laboratory of Darfur Universities, Mousseh District, 63311 Nyala, Sudan; 4 Faculty of Veterinary Science, University of Nyala, Mousseh District, 63311 Nyala, Sudan

**Keywords:** Levamisole efficacy, Gastrointestinal nematodes, Ruminants, South Darfur, Sudan

## Abstract

**Background:**

This study evaluated the efficacy of levamisole against gastrointestinal nematodes (GINs) in cattle, sheep and goats in five regions from South Darfur, Sudan, based on faecal egg count reduction tests (FECRTs) and coprocultures. Levamisole efficacy was examined in 45 cattle (8 mg/kg dose) from Bulbul, 22 sheep (8 mg/kg dose) from Nyala Domaia and 117 goats (10 and 12 mg/kg doses) from Bulbul (*n* = 25), Kass (*n* = 21), Nyala Domaia (*n* = 40) and Tulus (*n* = 31), all naturally infected with GINs. Moreover, the efficacy was evaluated in goats (*n* = 10) experimentally infected with a local *Haemonchus contortus* Um Dafuq isolate and treated with an 8 mg/kg dose. Faecal samples were collected before treatment (day 0) and day 12 after treatment for natural infection trials, while different collection times were used for experimentally infected goats.

**Results:**

A parametric FECRT model with individual efficacy and parametric and non-parametric models with common efficacy assigned levamisole susceptibility to strongyle nematodes in cattle. However, all three models classified strongyles in goats as resistant to 10 and 12 mg/kg in Bulbul, Kass and Nyala Domaia, with lower 90% confidence intervals (CIs) of 77.7 – 94.4% and upper 90% CIs of 86.6 – 97.5%. Discrepancies occurred between the models in sheep in Nyala Domaia and goats in Tulus. The experimental infection trial in goats supported findings from goat field trials. Host species and sex were significant factors in resistance (*P* < *0.05*). Using simulated data, a power analysis to detect resistance or susceptibility showed a power above 80.0% if at least 10 animals were used, overdispersion was low and the true efficacy was < 97.0% or > 99.2%, respectively. Model fit comparisons revealed that the individual efficacy eggCounts models fitted the data significantly (*P* < *0.05*) better than the common efficacy models. Mild cholinergic toxicity was recorded in one sheep and seven goats, particularly those that received the 12 mg/kg dose.

**Conclusions:**

The GINs of cattle were levamisole susceptible, whereas those of sheep and goats were resistant. Caution is advised with dose adjustments in desert sheep and goats. Research into combination anthelmintics and targeted selective treatment is recommended to address resistance in South Darfur.

**Supplementary Information:**

The online version contains supplementary material available at 10.1186/s12917-026-05320-2.

## Background

Helminth infections pose a significant global health problem for humans and animals [1]. In tropical regions, such as Sudan, helminth infections are more intense due to environmental conditions that favour the survival of exogenous stages of parasites [2, 3]. In animals, helminth infections are often subclinical. However, gastrointestinal nematodes (GINs) can cause clinical parasitic gastroenteritis, particularly in grazing ruminants, leading to significant economic losses, and the abomasal *Haemonchus* spp. have the strongest impact [4]. *Haemonchus contortus* primarily infects sheep and goats, while *Haemonchus placei* mainly affects cattle. Both species, however, can occasionally infect all three ruminants, indicating cross-infection [5, 6]. Other GIN parasites with a significant economic impact on ruminant production include the genera *Teladorsagia*, *Cooperia*, *Trichostrongylus*, *Chabertia*, *Oesophagostomum* and, to a lower level, *Strongyloides* [4, 7].

In veterinary and human medicine, control of GIN infections over the past four to six decades has mainly relied on three major anthelmintic classes: benzimidazoles (e.g. albendazole), imidazothiazoles (levamisole) and macrocyclic lactones (e.g. ivermectin) [7, 8]. However, prolonged and frequent use of drugs from these classes has led to widespread resistance in veterinary parasites [9]. The impact of anthelmintic resistance on ruminant production is substantial, particularly in industrial farming, with annual losses estimated at 38 million euros across 18 European countries [4]. These losses arise from persistent infections, repeated clinical diagnoses and the cost of anthelmintics [4, 9]. Recently, resistance has become an increasing issue in regions practising subsistence farming, with reports of multi-drug resistance from many African countries [10–12].

Levamisole is a broad-spectrum anthelmintic drug mainly active against nematodes and frequently used in veterinary medicine [13]. In addition to its anti-nematode effects, it also has immunomodulatory properties [14]. Levamisole induces spastic paralysis in susceptible nematodes by causing permanent muscle contraction due to activation of the nicotinic acetyl-choline receptor [15]. However, its narrow therapeutic index poses a significant drawback compared to other anthelmintics, as toxic levels may be reached at only four times the therapeutic dose, with reports indicating that toxicity may occur at even twice the therapeutic dose [16]. This risk is attributed to its nicotine-like structure and effects, making dose escalation an unfavourable option [17]. In goats, the bioavailability of levamisole, as well as other anthelmintics, is lower than in sheep. Therefore, to achieve in goats a similar plasma concentration as in sheep, at least a 1.5-fold higher dose than in sheep must be administered [18]. However, caution is advised due to the risk of neurological (cholinergic) toxicity. Levamisole resistance in GINs infecting cattle, sheep and goats has been reported in industrial farming in Australia [19], parts of Europe, including France, Greece, Germany and the UK, as well as in South America (particularly Brazil) [20, 21], South Africa [22], the United States [23] and in some countries in Asia [24–26]. In recent years, reports of levamisole resistance have been increasing in subsistence farming regions, indicating a wider resistance problem and more control challenges [11, 27, 28].

Knowing the efficacy of each anthelmintic drug across different animal species in specific regions is a crucial step in parasite control and in monitoring resistance development. The faecal egg count reduction test (FECRT) is described as the most suitable method for assessing the efficacy of all marketed anthelmintics, including levamisole [29]. In recent years, the Mini-FLOTAC technique has become an often-used method for the identification and counting of helminth eggs, particularly GINs, in ruminants due to its low egg count multiplication factor compared to the classical McMaster method protocol [30, 31].

In Sudan, livestock farming includes 30 million cattle, 40 million sheep and 31 million goats, making a significant contribution to the national Gross Domestic Product (GDP) [32]. In recent years, efforts have focused on understanding the situation of anthelmintic resistance in this country. Studies on benzimidazole resistance in cattle and goats in South Darfur detected widespread benzimidazole-resistant *H. contortus* in goats and, to some extent, in cattle [3, 6]. Recently, reduced ivermectin efficacy has been reported in sheep and goats, with resistance strongly associated again with *H. contortus*, as it survived at eight times the recommended ivermectin dose for sheep [33]. The objectives of this study were to evaluate the efficacy of levamisole in cattle, sheep and goats naturally infected with GINs in South Darfur, to assess levamisole efficacy in goats experimentally infected with *H. contortus* isolated from a local abattoir and to provide information on neurological toxicity, particularly in the local desert goat breed at different doses.

## Materials and methods

### Study location and design

This study was conducted across five regions in South Darfur (11.30°N, 24.40°E), southwestern Sudan: Bulbul (12.43°N, 24.29°E), Kass (12.50°N, 24.28°E), Nyala (12.05°N, 24.88°E), Tulus (11.00°N, 2.00°E) and Um Dafuq (10.41°N, 23.41°E). In Nyala, the capital of South Darfur, the western subregion Domaia was included. Further details on South Darfur, along with a map and information about these regions, are available in Mohammedsalih et al. [3]. South Darfur lies within a savannah zone, with land mostly covered by clay-sandy soil. The dominant grass on the pastures is abo-asabei (Egyptian crowfoot grass, *Dactyloctenium aegyptium*). South Darfur has a long dry season, compromising winter and summer, and a single rainy season from June to November, with precipitation ranging between 377 and 546 mm per month. The average minimum and maximum temperatures range between 24.7 °C and 37.6 °C, and relative humidity varies between 28.3% and 56.7% [32, 34].

The present study was primarily designed to evaluate the anthelmintic efficacy of levamisole in goats. Sheep and cattle were included only when available on farms within the logistical framework of the study; consequently, efficacy in these two species was not evaluated in all four selected regions.

This study was conducted between 2019 and 2022, during the rainy season (June – November), to evaluate levamisole efficacy in local breeds of cattle (*Bos taurus*, Bagara cattle), sheep (*Ovis aries*, desert sheep) and goats (*Capra hircus*, desert goats) naturally infected with GINs in Bulbul (autumn 2021 and 2022), Kass (autumn 2020), Nyala Domaia (autumn 2019) and Tulus (autumn 2020), based on FECRTs and coprocultures. Levamisole efficacy was also assessed in goats experimentally infected with *H. contortus* isolated from goat abomasa at the Um Dafuq abattoir (December 2020 – February 2021). In cattle and sheep, levamisole was evaluated at a recommended dose of 8 mg/kg body weight (bw) [18]. In goats, three doses were evaluated: 8, 10 and 12 mg/kg bw. In the four selected regions, farmers typically practice subsistence farming and keep animals in their houses (in small pens). Most of them have between 2 and 50 animals, but a few farms have larger numbers. Goats are the most predominant species; however, some farms rear goats with cattle, goats with sheep, and rarely, farms keep all three animal species. The three animal species typically share pastures in each region during the day and receive concentrated feed upon returning to their farms. This husbandry practice facilitates the sharing of the GIN populations between the animals in the pasture. Basically, for this study, and as we did in our previous studies on benzimidazole [3, 6] and ivermectin [33], levamisole efficacy was evaluated in the three animal species at the regional level.

### Levamisole treatment in cattle, sheep and goats naturally infected with gastrointestinal nematodes

In Bulbul, Kass, Nyala Domaia and Tulus, 26 farms (median: 6 animals; range: 4–25 heads, for each animal species) were visited to screen for GINs in cattle, sheep and goats. Farms were selected if they had more than four animals from any of the three species and no anthelmintic treatments in the 30 days before screening. It was not required to have all three animal types on a single farm, but each farm needed more than four animals, for each species, and none could be treated during the one-month study period. Due to study logistics, cattle were screened only in Bulbul on 10 farms, sheep only in Nyala Domaia on five farms and goats across all four regions: 10 farms in Bulbul (the same cattle farms), five in Kass, five in Nyala Domaia (the same sheep farms) and six in Tulus. On each farm, cattle, sheep and goats of both sexes and of different age groups (young: < 1 year; adult: > 1 years, based on dentition [35]) were examined for GINs infection using the Mini-FLOTAC method. Animals of each species shedding more than 150 strongyle eggs per gram (EPG) of faeces were selected for inclusion in the treatment trials [36]. Table 1 summarises the total number of male and female cattle, sheep and goats screened, grouped by age across the four regions and those included in the levamisole efficacy evaluation and available at the study’s end. A total of 45 cattle, 22 sheep and 117 goats were treated with levamisole and available at the study’s end. Before levamisole administration, each animal’s body weight was determined individually by measuring heart girth and body length [37] and calculated according to the following formula:

**Table 1:** Cattle, sheep and goats were used to assess the efficacy of levamisole across four different study regions in South Darfur, Sudan

Study animal	Factor	Total	Sex		Age		Study region			
			Male	Female	Young	Adult	Bulbul	Kass	Nyala Domaia	Tulus
Cattle	All tested cattle	96								
	No. of cattle assigned per category	n.a	44	52	88	8	96	n.a	n.a	n.a
	No. (%) of cattle shedding strongyle eggs	62 (64.6)	26 (59.1)	36 (69.2)	58 (65.9)	4 (50.0)	62 (64.6)	n.a	n.a	n.a
	No. (%) of cattle shedding > 150 strongyle eggs per gram faeces	57 (59.4)	25 (56.8)	32 (61.5)	54 (61.4)	3 (37.5)	57 (59.4)	n.a	n.a	n.a
	No. (%) of selected and treated cattle that completed the trial	45 (46.9)	17 (38.6)	28 (53.9)	43 (48.9)	2 (25.0)	45 (46.9)	n.a	n.a	n.a
Sheep	All tested sheep	37								
	No. of sheep assigned per category	n.a	5	32	9	28	n.a	n.a	37	n.a
	No. (%) of sheep shedding strongyle eggs	29 (78.4)	4 (80.0)	25 (78.1)	9 (100)	20 (71.4)	n.a	n.a	29 (78.4)	n.a
	No. (%) of sheep shedding > 150 strongyle eggs per gram faeces	28 (75.7)	4 (80.0)	24 (75.0)	8 (88.9)	20 (71.4)	n.a	n.a	28 (75.7)	n.a
	No. (%) of selected and treated sheep that completed the trial	22 (59.5)	4 (80.0)	18 (56.3)	6 (66.7)	16 (57.1)	n.a	n.a	22 (59.5)	n.a
Goats	All tested goats	170								
	No. of goats assigned per category	n.a	20	150	51	119	40	30	50	50
	No. (%) of goats shedding strongyle eggs	143 (84.1)	17 (85.0)	126 (84.0)	43 (84.3)	100 (84.0)	31 (77.5)	25 (83.3)	42 (84.0)	45 (90.0)
	No. (%) of goats shedding > 150 strongyle eggs per gram faeces	127 (74.7)	17 (85.0)	110 (73.3)	39 (76.5)	88 (74.0)	27 (67.5)	21 (70.0)	40 (80.0)	39 (78.0)
	No. (%) of selected and treated goats that completed the trial	117 (68.8)	14 (70.0)	103 (68.7)	31 (60.8)	86 (72.3)	25 (62.5)	21 (70.0)	40 (80.0)	31 (62.0)

$$\text{Heart girth }\times \text{heart girth }\times \text{body length}/300=\text{animal weight in pounds }\left(\text{then converted to kilograms}\right)$$

Levamisole was imported from Germany (levamisole 100 mg/ml Inj.-Lsg., cp-pharma, Burgdorf, Germany). Cattle (*n* = 45) and sheep (*n* = 22) received a single dose via subcutaneous (SC) injection at the recommended dose of 8 mg/kg bw [18]. In goats, 10 and 12 mg/kg bw doses were examined. The 1.5-fold levamisole dose recommended for sheep (12 mg/kg bw) administered SC had not been previously studied in Sudanese local goats (desert goats). In Nyala Domaia, this dose was examined in 10 naturally GIN-infected goats, comprising males and females of different ages. Body weight for these 10 goats was measured with a spring balance (100 kg limit) and doses were calculated accordingly. Levamisole efficacy at a dose of 10 mg/kg bw was evaluated in 107 goats across the four study regions. Faecal samples were collected on day 0 (pre-treatment) and day 12 post-treatment for all host species. All treated animals were monitored for six hours post-levamisole injection for signs of neurological toxicity [16]. Study animals remained on their farms throughout the study and afterwards.

### Levamisole treatment in goats experimentally infected with *Haemonchus contortus*

This experimental infection trial was designed to support the field trial findings, particularly for sheep and goats. *Haemonchus contortus* was found to be the most abundant GIN species implicated in anthelmintic resistance in South Darfur [3, 6, 33] and was therefore examined in this trial. Since increasing the levamisole dose poses risks due to its narrow therapeutic index [16], the trial examined the efficacy of levamisole at the standard Sudanese dose for goats (8 mg/kg bw) using *H. contortus* local isolate suspected to be levamisole susceptible. To increase the likelihood of obtaining a levamisole-susceptible *H. contortus* field isolate, a region in South Darfur with minimal levamisole use was identified. Discussions with veterinary drug distributors in Nyala (Al-Huda, AVICO and Bash Pharma) indicated that Um Dafuq was one of the regions in South Darfur with the lowest levamisole orders from veterinary pharmacies, making it a suitable option to collect the infective material. The trial was conducted on the premises of the Faculty of Veterinary Science, University of Nyala, Sudan (December 2020 – February 2021). Ten male goats under six months old were infected with *H. contortus* and treated with levamisole. The protocol followed Mohammedsalih et al. [3]: 50 abomasa from naturally *H. contortus* infected goats were collected at the Um Dafuq abattoir, with unknown history of anthelmintic treatments. Mature gravid *H. contortus* females were isolated, crushed, pooled and cultured in heat-treated bovine faeces. After 10 days of incubation (22–27 °C), infective third-stage larvae (L3) were harvested using the Baermann technique. Experimental goats were firstly confirmed helminth egg-free by examining the faecal samples using Mini-FLOTAC method [30], then purchased (Nyala livestock market), transported to the faculty premises and kept for 21 days with weekly faecal examinations. Throughout the study, goats were fed dry hay with free access to water and care was taken to prevent contamination from external nematode larvae. On day 21, after confirming negative helminth infection results, goats were weighed (median: 12 kg bw; range: 7–13 kg, using a spring balance (100 kg limit)), then inoculated orally with 150 L3 larvae/kg bw. From day 10 after inoculation, faecal samples were collected daily. On day 25, the expected peak of egg shedding [3, 33], goats were weighed again and treated SC with levamisole at 8 mg/kg bw. Faecal egg counts (FECs) were recorded pre-treatment (day 0) and on days 5, 8, 10, 12 and 14 post-levamisole administration.

### Faecal sample analyses

#### Faecal egg counts

Faecal samples were collected directly from the rectum of individual cattle, sheep and goats into labelled plastic bags and stored at 4 °C for up to 24 h before egg counting. Samples from the Bulbul, Nyala Domaia and Um Dafuq trials were analysed at the Laboratory of Parasitology, Faculty of Veterinary Science, University of Nyala, while those from Kass and Tulus were analysed directly in the field. Five grams of faeces from each sample were weighed using a sensitive balance (Shimadzu BL220H, Shimadzu Corporation, Kyoto, Japan), then homogenised in a pestle and mortar with 45 ml of sodium chloride solution (relative density: 1.20) and filtered through a tea strainer (mesh size ~ 0.3 mm) into a 400 ml glass measuring beaker. The filtered material was used to fill the two chambers of the Mini-FLOTAC device, then left for 10 min to allow helminth eggs to float [30]. Under a light microscope (Olympus CX23, Olympus Corporation, Shinjuku, Japan), nematode eggs were identified and counted using standard keys [38]. Counted eggs were expressed as EPG using a multiplication factor 5 for cattle and 10 for sheep and goats [30, 33].

#### Faecal cultures

Faecal samples were collected from cattle, sheep and goats in the natural infection trials pre-treatment (day 0) and on day 12 post-levamisole administration. Faecal samples from each farm were pooled by animal species after field collections, comprised at least four animals per pool. Faeces were mixed with wood shavings and incubated in labelled plastic jars at 22–27 °C, with daily moistening using distilled water for 10 days. Third-stage larvae were then isolated using the Baermann technique, identified under a light microscope, and the first 100 L3 (or all L3 if fewer than 100) were assigned to the genera *Haemonchus*, *Trichostrongylus* or the *Oesophagostomum*/*Chabertia* group using standard differentiation keys [38].

### Clinical scoring of levamisole toxicity

Levamisole toxicity was classified into mild, moderate and severe. Mild toxicity was considered to be present if one of the following symptoms occurred after treatment: restlessness, salivation, tail switching, tremors, frequent defecation, mild colic and jaw chomping. As signs of moderate toxicity, more severe cases of these clinical signs were observed. Severe toxicity was assumed if rapid respiration, hypersalivation, frothing at the mouth, muscle tremors, recumbency, paraesthesia, central nervous system depression or eventually death occurred [39].

### Statistical analyses

The data were analysed and interpreted according to the revised 2023 guideline on anthelmintic efficacy evaluation in ruminants from the World Association for the Advancement of Veterinary Parasitology (WAAVP) [40]. However, as this guideline had not been published at the time when the trials were conducted, the selection criteria for animals in treatment groups followed the original WAAVP guideline (a threshold of 150 EPG) [36]. However, all animal numbers were higher than recommended in the WAAVP guideline, given the multiplication factors of 5 (cattle) and 10 (sheep and goats) that were used and the mean EPG, which was above 1000 for all study groups. Based on the assumption of having at least 100 eggs seen for each animal (corresponding to the 1000 EPG with a multiplication factor of 10), a power analysis was conducted for the sensitivity and the resistance test for all three statistical methods, i.e. the delta method (default in bayescount) as well as eggCounts with common or individual efficacies. For this purpose, raw egg counts were simulated based on assumed negative binomial distribution with different overdispersion parameters k (k = 0.1, 0.3, 0.5, 0.75, 1 and 2). For the resistance test, assumed true efficacies of 0.8, 0.9, 0.96, 0.97, 0.98 and 0.99 were investigated, while the scenario for the power of the susceptibility test used assumed true efficacies of 0.99, 0.991, 0.992, 0.993, 0.994, 0.995, 0.997, 0.999 and 1. The number of animals included was varied over a wide range (N = 5, 10, 15, 20, 25, 30, 40 and 50). For each combination of k, N, and assumed true efficacy (288 for the resistance test, 432 for the susceptibility test, 1000 random egg count samples were generated (7.2 × 10^5^ egg count sample sets in total). All of these sets were then analysed with the delta (asymptotic) method (bayescount package version 1.2.1–2) and the eggCounts package version 2.5.1. The number of times a true efficacy > 0.99 in the susceptibility scenario or a true efficacy < 0.99 in the resistance scenario was actually detected were used to calculate the power of the tests for susceptibility and resistance. In addition, the frequency, how often a method was not able to calculate any 90% confidence intervals (CIs) and how often the result was inconclusive were determined. Power of resistance and susceptibility test, and frequencies were plotted as line plots using the number of animals for the x-axis and different graphs for different combinations of K and method. Plots were generated using ggplot2 version 4.0.0. Different true efficacies were plotted in the same graph using different colours. The simulation of egg count data and analyses with bayescount, as well as plotting of results, was conducted in RStudio using R-4.5.1, while R-4.2.2 on a high-performance computer (HPC) was used [41]. All R code and the Slurm scripts to start the R scripts and manage the HPC resources are available in Additional file 1. The R code for this purpose was optimised with the help of artificial intelligence (ChatGPT 5.1).

Three different statistical approaches were compared to analyse the paired data: (i) a parametric model with individual efficacy for each animal, (ii) a parametric model with common efficacy for all animals and (iii) a non-parametric model with common efficacy for all animals. No zero inflation was assumed for all models. Parametric models were calculated with the eggCounts package 2.3–3, while the non-parametric model was obtained from an analysis with bayescount package 1.1.0. For all models, the 90% CIs of faecal egg count reductions (FECRs) were calculated. The eggCount package also provides an estimate for the FECR, which is not the case for the bayescount package, and therefore, the mean reduction was used as an estimate in some figures. For eggCounts and the individual efficacy, the median and the 5% and 95% quantiles of the posterior distribution were used as estimates for FECR and its 90% CI. For the model with common treatment efficacy for all animals, the mode of the posterior distribution and the 90% highest posterior density interval were used as estimates for FECR and its 90% CI as suggested by Wang et al. [42]. All calculations with eggCounts were made using the web interface http://shiny.math.uzh.ch/user/furrer/shinyas/shiny-eggCounts/. The bayescount package reports 90% CI calculated using the delta method as detailed by Levecke et al. [43]. When the 90% CI could not be calculated using the bayescount since all post-treatment FECs were zero, we moved to the alternative suggestion, the parametric Beta Negative Binomial (BNB) method version A [44] to assign a susceptibility/resistance status. However, BNB version A was chosen as our treated animals in each trial was equal to or greater than 5, besides the zero post-treatment EPG. The BNB method version A assigned resistance (resistance test *P* < *0.001*; susceptibility test *P* = *1.000*), this calculation provided using the web interface https://www.fecrt.com.

The FECR results were interpreted as follows: a parasite community was assigned as susceptible when the upper 90% confidence limit (CL) was equal to or higher than 99% and the lower 90% CL was equal to or higher than 95%. Resistance was identified when the upper 90% CL was less than 99%. Low resistance (within the resistant status) was indicated when the upper 90% CL was less than 99% and the lower 90% CL was equal to or higher than 95%. A parasite community was assigned as inconclusive when the lower 90% CL was less than 95% and the upper 90% CL was higher than 99% [40].

To assess agreement between the three statistical models assigning FECR results, Cohen’s κ statistics were used, employing the CohenKappa() function from the DescTools package version 0.99.55 in R version 4.4.2 within RStudio version 2024.09.1, with agreement levels classified as no agreement (≤ 0), poor (0.01–0.20), fair (0.21–0.40), moderate (0.41–0.60), substantial (0.61–0.80) or almost perfect (≥ 0.81) [45].

For all FECRTs, the fit of the models with common and individual efficacy calculated with eggCounts was compared using K-fold cross-validation. After using the saveAll option of fecrt_stan(), the saved latent intensity parameters were used to construct log-likelihood matrices for each observation. Since, in a few cases, eggCounts gave out warnings that there were divergent transitions in the Markov Chain Monte Carlo (MCMC), combined with the suggestion to increase the adaptDelta parameter from its default of 0.95, all models were compared using adaptDelta values of 0.95 and 0.99. This increases robustness of posterior sampling and reduces problems with divergent transitions by forcing stan to use smaller and more accurate step sizes in the MCMC chains without changing the model itself. The script uses three cross-validation methods, i.e. K-fold cross-validation, Pareto-smoothed importance sampling leave-one-out (PSIS-LOO) and the Watanabe–Akaike Information Criterion (WAIC) using the loo package version 2.8.0. For all methods, expected log pointwise densities (elpd values) with standard deviation (SD) values were calculated. Models with higher elpd should be preferred. Final interpretation was based on K-fold cross-validation, and 95% CIs for elpd values were obtained using a bootstrapping approach. Finally, the elpd of the individual efficacy model was subtracted from the elpd of the common efficacy model, and the 95% CI of this difference was calculated. If the 95% CI did not include the value 0, a *P* < *0.05*% between the model fits was assumed. A positive difference with the lower confidence limit (LCL) > 0 was interpreted as a significantly better fit of the individual efficacy model compared to the common efficacy model. In contrast, a negative difference with the upper confidence limit (UCL) < 0 would indicate a better fit of the common efficacy model. The R code for this purpose was optimised with the help of artificial intelligence (ChatGPT 5.1) and is available for all FECRTs in Additional file 2.

Levamisole efficacy was evaluated separately for parasite communities across host species, regions and doses, as follows. The host factor was calculated first between animals in the same regions (cattle and goats in Bulbul; sheep and goats in Nyala Domaia) and then between all three animal species at the state level. However, the effects of region and levamisole dose were calculated only for goats, as trials with natural GIN infections in goats used two different doses and were conducted in all four study regions. Individual faecal egg count reductions (FECRi) for strongyles were calculated for each animal using the formula [37] below:

$$\mathrm{FECRi}\left(\mathrm{\%}\right)=100\times\left[1-\left({\mathrm{T}}_\mathrm{i}/{\mathrm{T}}_0\right)\right]$$

In the formula, T_i_ represents the EPG post-treatment and T_0_ represents the EPG pre-treatment (day 0). Initially, EPG data were collected, stored in Microsoft Excel and the formula was applied for FECRi calculation. The obtained FECRi values were subsequently transformed into binomial variables (FECRi99) using a threshold for full susceptibility (equal to or higher than 99% efficacy) as assigned as a cut-off for expected efficacy in the revised 2023 WAAVP guideline [40], where all FECRi values ≥ 99% were set to 0 and those below the threshold were set to 1. The extracted data were then used to perform logistic regression analyses with the following explanatory variables: (i) study region (based on goat data only): Tulus (reference) *vs.* Bulbul, Kass and Nyala Domaia. (ii) Host species in Bulbul: cattle *vs.* goats. (iii) Host species in Nyala Domaia: sheep *vs.* goats. (iv) Host species across all regions: cattle *vs.* sheep and goats. (v) Levamisole dose in goats in Nyala Domaia (where the 12 mg levamisole/kg bw was evaluated): 12 mg/kg bw *vs.* 10 mg/kg bw. (vi) Levamisole dose in goats across all four study regions: 12 mg/kg bw *vs.* 10 mg/kg bw. Bivariable model calculated for the explanatory variables (i), (ii), (iii) and (v) using the glm() function in R. The Akaike Information Criterion (AIC) was obtained using the drop1() function, the 95% CIs were calculated with confint() and odds ratios were derived by applying exp() to the estimates and CLs. Pseudo R^2^ values were computed using the RsqGLM() function from the modEvA package version 3.39, following Nagelkerke and Tjur’s methods. The explanatory variables host species in all regions (iv) and levamisole dose in goats across all regions (vi) were calculated using a generalized linear mixed model (GLMM) analysis (bivariable analysis) (glmmTMB package version 1.1.10 in R). Study region was included as a random effect. Odds ratios with 95% CIs were estimated using tidy() function (broom.mixed package version 0.2.9.6). The goodness-of-fit was assessed using the conditional R^2^ from the performance package version 0.14.0. The R script is available in Additional file 3.

In addition, a beta regression was conducted using the actual FECRi values (here scaled from 0 to 1) as dependent variables and the same set of independent variables using glmmTMB version 1.1.13. Comparison of model residues *vs.* simulated residues as implemented in the DHARMa package version 0.4.7 and the AIC were used to identify the best models. Initially, host species, sex and age group were considered as fixed variables and the study region as a random effect variable. For the dispersion formula, all independent variables were included. The R script was optimised using ChatGPT 5.1 and is available in Additional file 4.

In all analyses, a value of *P* < *0.05* was considered statistically significant.

## Results

### Levamisole treatment in cattle, sheep and goats naturally infected with gastrointestinal nematodes

In the three study animal species, eleven FECRTs were conducted in the four selected study regions of South Darfur: Bulbul (cattle: two FECRT tests; goats: two tests), Kass (goats: two tests), Nyala Domaia (sheep: one test; goats: three tests) and Tulus (goats: one test). Raw data for all samples can be found in Additional file 5: Table S1 and Additional file 6: Table S2. Of the eleven FECRTs, seven assessed efficacy against strongyle nematodes, which were found to be fully compliant with the revised 2023 WAAVP guideline, meeting the required number of animals per trial (Tables 2, 3 and 4) and the FECs, while four targeted *Strongyloides papillosus* (Tables 2, 3 and 4).

**Table 2:** Results of faecal egg count reduction (FECR) with 90% confidence intervals (CIs) in cattle naturally infected with gastrointestinal nematodes, before and after subcutaneous injection of levamisole (paired study design) at a therapeutic dose of 8 mg/kg body weight in Bulbul, South Darfur, Sudan

GI nematode	No. of cattle	Mean faecal egg counts (EPG)		Calculated sample size^a^	Sample size outcome^b^	FECR% (90% CI)			Test outcome^d^
		Day 0	Day 12			Parametric model with individual efficacy (eggCounts)^c^	Parametric model with common efficacy (eggCounts)^c^	Non-parametric model with common efficacy (bayescount)	
Strongyles	45	1044	13	12	Appropriate	100 (100–100)	98.8 (98.6–99.0)	(97.9–99.5)	S – S – S
*Strongyloides papillosus*	5	256	0			99.8 (98.3–100)	99.9 (98.8–100)	(Uncalculatable)	S – S – S

*EPG* eggs per gram faeces, *GI* gastrointestinal

^a^Calculated sample size for a faecal egg count reduction test as per the revised 2023 WAAVP guideline [40], obtained using bayescount package version 1.1.0 (https://www.fecrt.com)

^b^A trial is considered appropriate when the number of animals in each treatment group meets or exceeds the calculated sample size [40]

^c^FECRs were paired calculated by comparing data post and pre-treatment without zero-inflation option

^d^The outcome of FECRs were identified as resistant (R), low resistant (L), susceptible (S) or inconclusive (I) [40]. The results are presented in the order parametric model with individual efficacy, parametric model with common efficacy and non-parametric model with common efficacy. When the 90% CI could not be calculated using a non-parametric model with common efficacy due to post-treatment faecal egg counts of zero (Uncalculatable), the parametric Beta Negative Binomial (BNB) method version A [44] was used instead to assign a susceptible status

**Fig. 1:**
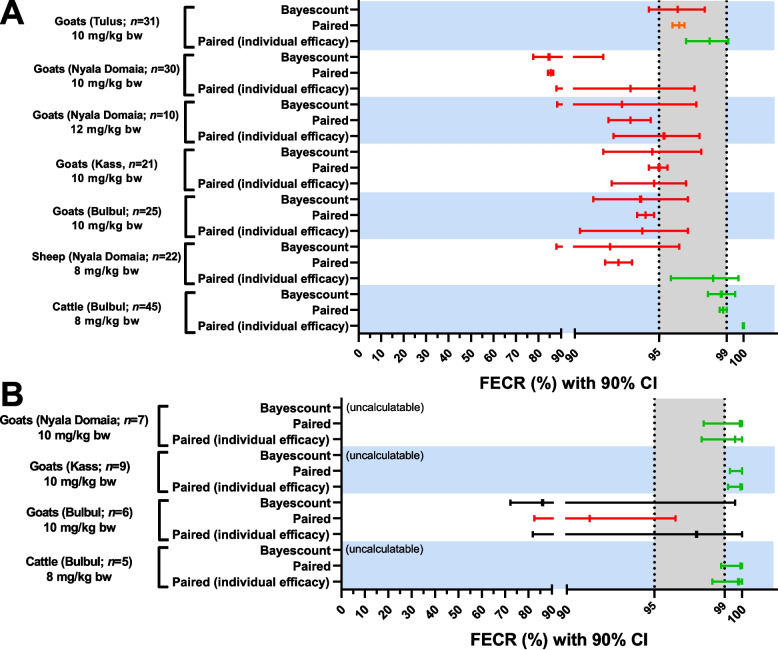
Results of faecal egg count reduction with 90% confidence intervals in cattle, sheep and goats naturally infected with gastrointestinal nematodes (strongyles (**A**) and *Strongyloides papillosus* (**B**)) before and after subcutaneous injection of levamisole at therapeutic doses across four different study regions in South Darfur, Sudan. Faecal egg counts (FECs) (paired study design) were analysed using a parametric model with individual efficacy for each animal (Paired (individual efficacy)), a parametric model with common efficacy for all animals (Paired) and a non-parametric model with common efficacy for all animals (Bayescount). No zero inflation was assumed across the three models. For the bayescount, the average reduction in FEC after treatment was calculated separately. Results were assigned to the status susceptible (green colour), low resistance (orange), resistance (red) or inconclusive (black) as recommended in the revised 2023 WAAVP guideline [40]. The 90% CI could not be calculated for *S. papillosus* using bayescount due to post-treatment zero FEC (uncalculatable). The grey zone indicates the range between the lower efficacy target of 95% and the expected efficacy of 99%. Abbreviations: bw, body weight; CI, confidence interval; FECR, faecal egg count reduction

In cattle in Bulbul, levamisole was effective across all statistical analysis methods for both strongyles and *S. papillosus*. For *S. papillosus*, the bayescount method was unable to calculate a 90% CI since post-treatment EPG values were zero for all animals. The BNB version A method was chosen by bayescount instead to assign the population a susceptible status (Table 2; Fig. 1A and B). No toxicity of levamisole was observed in cattle (*n* = 45).

In sheep from Nyala Domaia, levamisole efficacy against strongyles was reduced according to the parametric and non-parametric models with common efficacy in all animals, while the analysis based on a parametric model with individual efficacy for each animal concluded susceptibility in strongyle communities in Nyala Domaia (Table 3; Fig. 1A). Mild cholinergic toxicity was observed in 4.6% (1/22) of the sheep at the recommended dose, and symptoms lasted for 30 min without antagonistic treatment.

**Table 3:** Results of faecal egg count reduction (FECR) with 90% confidence intervals (CIs) in sheep naturally infected with gastrointestinal nematodes, before and after subcutaneous injection of levamisole (paired study design) at a therapeutic dose of 8 mg/kg body weight in Nyala (Domaia), South Darfur, Sudan

GI nematode	No. of sheep	Mean faecal egg counts (EPG)		Calculated sample size^a^	Sample size outcome^b^	FECR% (90% CI)			Test outcome^d^
		Day 0	Day 12			Parametric model with individual efficacy (eggCounts)^c^	Parametric model with common efficacy (eggCounts)^c^	Non-parametric model with common efficacy(bayescount)	
Strongyles	22	2186	162	7	Appropriate	98.2 (95.7–99.7)	92.6 (91.8–93.4)	(88.0–96.2)	S – R – R

*EPG* eggs per gram faeces, *GI* gastrointestinal

^a^Calculated sample size for a faecal egg count reduction test as per the revised 2023 WAAVP guideline [40], obtained using bayescount package version 1.1.0 (https://www.fecrt.com)

^b^A trial is considered appropriate when the number of animals in each treatment group meets or exceeds the calculated sample size [40]

^c^FECRs were paired calculated by comparing data post and pre-treatment without zero-inflation option

^d^The outcome of FECRs were identified as resistant (R), low resistant (L), susceptible (S) or inconclusive (I) [40]. The results are presented in the order parametric model with individual efficacy, parametric model with common efficacy and non-parametric model with common efficacy. When the 90% CI could not be calculated using a non-parametric model with common efficacy due to post-treatment faecal egg counts of zero (Uncalculatable), the parametric Beta Negative Binomial (BNB) method version A [44] was used instead to assign a susceptible status

In goats, the 12 mg/kg bw dose was assessed in 10 animals for efficacy and toxicity in Nyala Domaia. Despite this high dose, strongyle nematodes were identified as levamisole resistant with all three statistical models (Table 4; Fig. 1A). However, 40.0% of the goats that received the 12 mg/kg bw dose exhibited mild cholinergic toxicity within 30 min after SC injection (see Additional file 7: Video S1). Recovery began between 30 min and an hour after treatment, and the symptoms were no longer observed after two hours. No deaths were recorded, and no animals required treatment for toxicity. Consequently, to prevent disagreement of the farmers regarding the continuation of the trials considering the animal welfare issues, the subsequent trials were conducted using a 10 mg/kg bw dose. Levamisole efficacy against strongyles in goats was clearly reduced in three out of four regions, with resistance identified in Bulbul, Kass and Nyala Domaia by all three statistical models (Table 4; Fig. 1A). Interpretation of efficacy data in Tulus showed discrepancies as the non-parametric model with common efficacy assigned resistance to the strongyle nematodes, parametric model with common efficacy assigned low resistance, while the parametric model with individual efficacy for each animal classified these parasites as susceptible (Table 4; Fig. 1A). At a dose of 10 mg/kg bw, 2.8% of the goats (3/107) displayed cholinergic toxicity, but the symptoms were milder (hyperactivity and slight muscle tremors) than those observed in goats treated with 12 mg/kg bw, and they lasted only for up to 30 min without treatment for toxicity.

**Table 4:** Results of faecal egg count reduction (FECR) with 90% confidence intervals (CIs) in goats naturally infected with gastrointestinal nematodes, before and after subcutaneous injection of levamisole (paired study design) at different doses across four study regions in South Darfur, Sudan

Study region	GI nematode	Dose (mg/kg bw)	No. of goats in each trial	Mean faecal egg counts (EPG)		Calculated sample size^a^	Sample size outcome^b^	FECR% (90% CI)			Test outcome^d^
				Day 0	Day 12			Parametric model with individual efficacy (eggCounts)^c^	Parametric model with common efficacy (eggCounts)^c^	Non-parametric model with common efficacy(bayescount)	
Bulbul	Strongyles	10	25	3203	186	5	Appropriate	94.0 (90.3–96.7)	94.2 (93.7–94.7)	(91.1–96.7)	R – R – R
	*Strongyloides papillosus*		6	147	13			97.4 (81.8–100)	91.3 (82.5–96.2)	(72.3–99.6)	I – R – I
Kass	Strongyles	10	21	2894	146	5	Appropriate	94.7 (92.2–96.6)	95.0 (94.4–95.5)	(91.7–97.5)	R – R – R
	*S. papillosus*		9	450	0			99.9 (99.2–100)	100 (99.3–100)	(Uncalculatable)	S – S – S
Nyala Domaia	Strongyles	12	10	1772	117	5	Appropriate	95.3 (92.3–97.4)	93.3 (92.0–94.5)	(88.3–97.2)	R – R – R
	Strongyles	10	30	1465	213	5	Appropriate	93.3 (88.0–97.1)	85.5 (84.2–86.6)	(77.7–91.7)	R – R – R
	*S. papillosus*		7	197	0			99.6 (97.7–100)	99.9 (97.8–100)	(Uncalculatable)	S – S – S
Tulus	Strongyles	10	31	3606	138	5	Appropriate	98.0 (96.6–99.1)	96.2 (95.8–96.5)	(94.4–97.7)	S – L – R

*bw* Body weight, *EPG* Eggs per gram faeces, *GI* Gastrointestinal

^a^Calculated sample size for a faecal egg count reduction test as per the revised 2023 WAAVP guideline [40], obtained using bayescount package version 1.1.0 (https://www.fecrt.com)

^b^A trial is considered appropriate when the number of animals in each treatment group meets or exceeds the calculated sample size [40]

^c^FECRs were paired calculated by comparing data post and pre-treatment without zero-inflation option

^d^The outcome of FECRs were identified as resistant (R), low resistant (L), susceptible (S) or inconclusive (I) [40]. The results are presented in the order parametric model with individual efficacy, parametric model with common efficacy and non-parametric model with common efficacy. When the 90% CI could not be calculated using a non-parametric model with common efficacy due to post-treatment faecal egg counts of zero (Uncalculatable), the parametric Beta Negative Binomial (BNB) method version A [44] was used instead to assign a susceptible status

*Strongyloides papillosus* eggs were not detected in sheep faecal samples, but they were found in goats in Bulbul, Kass and Nyala Domaia. The revised 2023 WAAVP guideline for the FECRT did not mention *Strongyloides* spp. Therefore, all selected goats that were positive for *S. papillosus* were used to evaluate levamisole efficacy against the parasite, without a *S. papillosus* cut-off EPG criterion. In Nyala Domaia and Kass, no *S. papillosus* eggs were detected after treatment, so the bayescount method could not calculate a 90% CI. The BNB method version A was chosen by bayescount instead, assigning the population a susceptible status. Moreover, both eggCount models used also assigned levamisole susceptibility. In Bulbul, discrepancies between the models were detected (Table 4; Fig. 1B).

Pre-treatment coprocultures of cattle, sheep and goats showed mixed infections with strongyle L3 larvae, including *Haemonchus* spp., *Trichostrongylus* spp. and *Oesophagostomum* spp./*Chabertia* spp. (Chabertiidae), with *Haemonchus* spp. being the most abundant (71.0–89.0%) (Table 5). Post-treatment, faecal cultures were not successful in cattle due to low egg counts, while only *Haemonchus* spp. larvae were identified in sheep and goats after treatment.

**Table 5:** Coprocultures of gastrointestinal helminths in the faeces of naturally infected cattle, sheep and goats were conducted before treatment (day 0) and 12 days after levamisole administration across four different study regions in South Darfur, Sudan

Coprocultures for strongyles (%)	Bulbul				Kass		Nyala, Domaia						Tulus	
	Cattle^a^		Goats^b^		Goats^b^		Sheep^a^		Goats^b^		Goats^c^		Goats^b^	
	Day 0	Day 12^d^	Day 0	Day 12	Day 0	Day 12	Day 0	Day 12	Day 0	Day 12	Day 0	Day 12	Day 0	Day 12
*Haemonchus* spp.	71	n.a	79	100	82	100	74	100	78	100	72	100	89	100
*Trichostrongylus* spp.	26	n.a	14	0	14	0	20	0	15	0	18	0	11	0
*Oesophagostomum* spp.*/Chabertia* spp.	3	n.a	7	0	4	0	6	0	7	0	10	0	0	0

*n.a.* Not available

^a^Cattle and sheep were used to evaluate levamisole efficacy at a dose of 8 mg/kg body weight (bw)

^b^Goats were used to evaluate levamisole efficacy at a dose of 10 mg/kg bw

^c^Goats were used to evaluate levamisole efficacy at a dose of 12 mg/kg bw

^d^Faecal cultures were unsuccessful due to low egg counts

### Factors associated with levamisole efficacy in cattle, sheep and goats

The explanatory variables host species, study regions and levamisole dose were analysed in cattle, sheep and goats naturally infected with strongyle nematodes, using the binary FECRi99 as the dependent variable in a bivariable logistic regression model and a GLMM (Tables 6 and 7). In Bulbul, the host species (cattle *vs.* goats) was significantly (odds ratio: 21.714; 95% CI: 6.580–85.572; *P* < *0.001*) associated with levamisole resistance (Table 6). However, analysing data for cattle, sheep and goats across all four regions is not fully representative, as all three host species were not included in each region. Nevertheless, this analysis provides valuable insights into the resistance status of GIN communities in the examined animals. Based on GLMM analysis, resistance to levamisole in South Darfur was significantly associated with strongyle nematodes infecting sheep (odds ratio: 5.429; 95% CI: 1.700–17.337; *P* = *0.004*) and goats (odds ratio: 13.818; 95% CI: 5.612–34.025; *P* < *0.001*). The study region was included as a random effect variable (Table 7).

**Table 6:** Bivariable logistic regression to identify variables affecting the odds of levamisole treatment efficacy to be < 99% in individual animals naturally infected with strongyle nematodes across host species and four study regions in South Darfur, Sudan

Variable	Estimate	Standard error	Odds ratio	95% confidence interval	*P* value	Nagelkerke	Tjur’s D	AIC
Intercept	0.460	0.369	1.583	0.778–3.354	0.213			
Study region (goats only): Tulus* vs*						0.042	0.030	141.200
Bulbul	0.927	0.621	2.526	0.777–9.213	0.136			
Kass	0.987	0.667	2.684	0.769–11.065	0.139			
Nyala Domaia	0.388	0.505	1.474	0.546–4.006	0.443			
Intercept	−1.692	0.411	0.184	0.075–0.387	< 0.001			
Host species (Bulbul only): Cattle *vs*						0.466	0.402	95.351
Goats	3.078	0.647	21.714	6.580–85.572	< 0.001			
Intercept	0.000	0.426	1.000	0.428–2.335	1.000			
Host species (Nyala Domaia only): Sheep *vs*						0.052	0.039	83.774
Goats	0.847	0.549	2.333	0.800–6.974	0.122			
Intercept	0.847	0.690	2.333	0.649–10.827	0.220			
Levamisole dose in goats (Nyala Domaia only): 12 mg/kg body weight *vs*						0.000	0.000	50.869
10 mg/kg body weight	0.000	0.797	1.000	0.184–4.560	1.000

**Table 7:** Final logistic regression mixed model to identify factors influencing the odds of levamisole treatment efficacy to be < 99% in individual animals naturally infected with strongyle nematodes in different host species in South Darfur, Sudan, using the study region (Bulbul, Kass, Nyala (Domaia) and Tulus) as a random effect variable

Variable	Estimate	Standard error	Odds ratio	95% confidence interval	*P* value	Conditional R^2^	Marginal R^2^
Intercept	−1.692	0.411	0.184	0.082–0.413	< 0.001		
Host species: Cattle *vs*						0.272	0.272
Sheep	1.692	0.592	5.429	1.700–17.337	0.004		
Goats	2.626	0.460	13.818	5.612–34.025	< 0.001		
Intercept	0.847	0.690	2.333	0.603–9.023	0.220		
Levamisole dose in goats: 12 mg/kg body weight *vs*						0.000	0.000
10 mg/kg body weight	0.095	0.723	1.100	0.267–4.536	0.895		

**Fig. 2:**
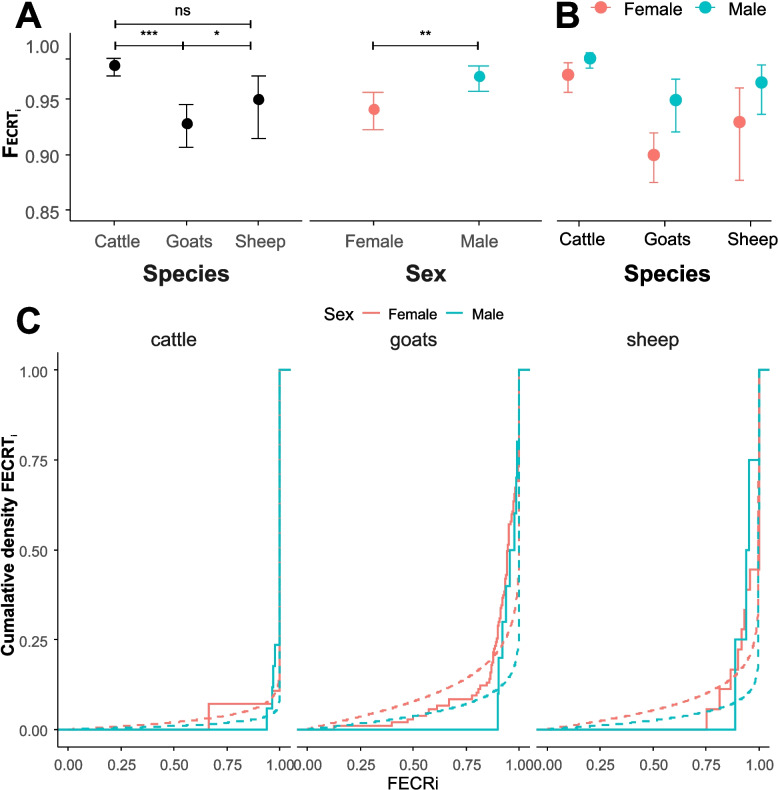
Variables with influence on individual faecal egg count reduction (FECRi, scale 0–1) identified by beta-regression. **A** Effects of host species and sex are shown. **B** Data are further split for each combination on host species and sex. No further statistical analysis was used here. The data and the model are the same as in (A). **C** Cumulative density plots showing as histogram the distribution of FECRi for each combination of host species and sex and the beta-regression model as broken lines fitted for these values

In order to test the effect of the variables sex, age group and host species on the actual FECRi of individual animals, a beta regression was performed. Initially, the region was included as a random effect variable. After model optimisation, primarily based on AIC values, the final model only included the fixed effect variables host species and sex. Figure 2A shows that the final multi-variable model includes only the variables sex and host species. Multi-comparison analysis of the data shows that there was a significantly lower efficacy in sheep and goats compared to cattle, and also female animals showed significantly lower FECRi values than males. In Fig. 2B, the combined effects of host species and sex are shown. No statistical analysis was conducted for this comparison. The data are from the same analysis as in part (A). As seen in Additional file 8: Table S3, the female cattle have a very high precision φ, indicating a low dispersal of the data. In contrast, dispersion was significantly higher (smaller precision φ) in goats than in cattle and in males than in females. Figure 2C shows model fit based on comparison of cumulative predicted and actual FECRi data.

### Levamisole treatment in goats experimentally infected with *Haemonchus contortus*

**Fig. 3:**
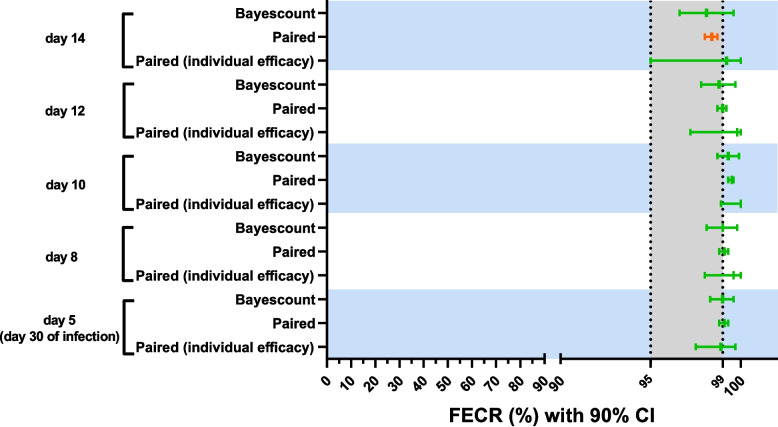
Results of faecal egg count reduction with 90% confidence intervals with male goats (*n* = 10) experimentally infected with *Haemonchus contortus* Um Dafuq isolate, South Darfur, Sudan, before and after a subcutaneous injection of 8 mg/kg body weight levamisole on day 25 post-infection. Faecal egg counts (FECs) (paired study design) were analysed using a parametric model with individual efficacy for each animal (Paired (individual efficacy)), a parametric model with common efficacy for all animals (Paired) and a non-parametric model with common efficacy for all animals (Bayescount). No zero inflation was assumed across the three models. For the bayescount, the average reduction in FEC after treatment was calculated separately. Results were assigned to the status susceptible (green colour), low resistance (orange), resistance (red) or inconclusive (black) as recommended in the revised 2023 WAAVP guideline [40]. The grey zone indicates the range between the lower efficacy target of 95% and the expected efficacy of 99%. Abbreviations: CI, confidence interval; FECR, faecal egg count reduction

The efficacy of levamisole in sheep and goats was reduced in strongyle nematodes in Bulbul (goats), Kass (goats), Nyala Domaia (sheep and goats) and Tulus (goats). To enhance the power of field trial findings in Sudan, locally recommended doses of levamisole in sheep and goats (8 mg/kg bw), as well as 10 and 12 mg/kg bw doses in goats, one option was to test levamisole efficacy at a higher dose, e.g. twice the recommended dose. This approach was constrained by levamisole’s narrow therapeutic index, which ranges between two to four times the recommended dose depending on the host species [16, 17]. Therefore, we applied an alternative strategy by evaluating levamisole efficacy in goats experimentally infected with a local nematode isolate collected from the abattoir of Um Dafuq region, which had the lowest levamisole orders according to veterinary drug distributors in South Darfur. This was done to determine whether the change in levamisole efficacy was due to resistance or other potential factors. This is particularly relevant for *H. contortus*, which is the most abundant GIN species involved in anthelmintic resistance evolution in sheep and goats in South Darfur [3, 33]. A local isolate of *H. contortus* was established by collection of female worms from goat abomasa at the Um Dafuq abattoir. Male goats were experimentally infected with L3 obtained from this isolate and treated SC with levamisole at 8 mg/kg bw, the standard levamisole dose for both sheep and goats in Sudan. Efficacy was evaluated at multiple sampling times. *Haemonchus contortus* eggs were first detected on day 16 post-L3 inoculation, and by day 18, all goats tested positive. Levamisole was administered on day 25, the expected peak of egg shedding. Levamisole at 8 mg/kg bw (the recommended dose for sheep and goats in Sudan) proved fully effective in goats experimentally infected with the *H. contortus* isolate from Um Dafuq, despite the fact that higher doses are internationally recommended for goats. A parametric model with individual efficacy for each animal and a non-parametric model with common efficacy for all animals assigned a susceptible status at different collection times after treatment. However, a parametric model with common efficacy for all animals assigned a susceptible status for days 5, 8, 10 and 12, while low resistance was suggested for the data from day 14 (Table 8; Fig. 3). Raw data for all samples can be found in Additional file 5: Table S1. None of the ten goats exhibited toxicity symptoms following levamisole administration.

**Table 8:** Faecal egg count reductions (FECRs) with 90% confidence intervals (CIs) in male goats (*n* = 10) experimentally infected with *Haemonchus contortus* isolates from goat abomasa at the Um Dafuq abattoir, South Darfur, Sudan, before and after a subcutaneous injection of 8 mg/kg body weight levamisole on day 25 post-infection

Day of sample collection (post-treatment)	Total eggs counted (Pre-treatment)	Mean faecal egg counts (eggs per gram)		Calculated sample size^a^	Sample size outcome^b^	FECR (%) (90% CI)			Test outcome^d^
		Pre-treatment	Post-treatment			Parametric model with individual efficacy (eggCounts)^c^	Parametric model with common efficacy (eggCounts)^c^	Non-parametric model with common efficacy (bayescount)	
5	6148	6148	56	5	Appropriate	98.9 (97.5–99.7)	99.1 (98.8–99.3)	(98.3–99.6)	S – S – S
8	6148	6148	56			99.6 (98.0–100)	99.1 (98.8–99.3)	(98.1–99.8)	S – S – S
10	6148	6148	32			100 (98.9–100)	99.5 (99.3–99.6)	(98.7–99.9)	S – S – S
12	6148	6148	64			99.8 (97.2–100)	99.0 (98.7–99.2)	(97.8–99.7)	S – S – S
14	6148	6148	100			99.2 (95.0–100)	98.4 (98.0–98.7)	(96.6–99.6)	S – L – S

^a^Calculated sample size for a faecal egg count reduction test as per the revised 2023 WAAVP guideline [40], obtained using bayescount package version 1.1.0 (https://www.fecrt.com)

^b^A trial is considered appropriate when the number of animals in each treatment group meets or exceeds the calculated sample size [40]

^c^FECRs were paired calculated by comparing data post and pre-treatment without zero-inflation option

^d^The outcome of FECRs were identified as resistant (R), low resistant (L), susceptible (S) or inconclusive (I) [40]. The results are presented in the order parametric model with individual efficacy, parametric model with common efficacy and non-parametric model with common efficacy

### Statistical models agreement

The FEC data, comprising paired pre- and post-treatment results following levamisole administration in cattle, sheep and goats naturally infected with GINs, as well as in goats experimentally infected with *H. contortus* across five regions in South Darfur, were analysed using three statistical models to assign GINs to one of the four categories outlined in the revised 2023 WAAVP guideline. Cohen’s κ analyses were conducted to determine the degree of agreement among the different statistical models (Table 9). Substantial agreements were observed between the eggCounts parametric model with individual efficacy for each animal and a non-parametric bayescount method with common efficacy for all animals (Cohen’s κ coefficient: 0.758, 95% CI: 0.447–1.069), as well as between the parametric eggCounts method and non-parametric bayescount with common efficacy (Cohen’s κ coefficient: 0.765, 95% CI: 0.484–1.045). However, the analysis of the parametric models with individual and common efficacy resulted in a Cohen’s κ coefficient of 0.529 (95% CI: 0.196–0.863), corresponding to a moderate agreement (Table 9).

**Table 9:** Agreement among three statistical analysis models used to assign trials in cattle, sheep and goats naturally infected with gastrointestinal nematodes and goats experimentally infected with *Haemonchus contortus*, after levamisole treatments in five different regions in South Darfur, Sudan

		**Parametric model with individual efficacy (eggCounts)**			**Parametric model with common efficacy (eggCounts)**			**Non-parametric model with common efficacy (bayescount)**			**Cohen’s κ (95% confidence interval)**	
		S	R	I	S	R	I	S	R	I	Parametric model with individual efficacy (eggCounts)	Parametric model with common efficacy (eggCounts)
Parametric model with individual efficacy (eggCounts)	S	11	n.a	n.a	8	3	0	9	2	0		
	R	n.a	4	n.a	0	4	0	0	4	0		
	I	n.a	n.a	1	0	1	0	0	0	1		
Parametric model with common efficacy (eggCounts)	S	8	0	0	8	n.a	n.a	8	0	0		
	R	3	4	1	n.a	8	n.a	1	6	1	0.529 (0.196–0.863)	
	I	0	0	0	n.a	n.a	0	0	0	0		
Non-parametric model with common efficacy (bayescount)	S	9	0	0	8	1	0	9	n.a	n.a		
	R	2	4	0	0	6	0	n.a	6	n.a	0.758 (0.447–1.069)	0.765 (0.484–1.045)
	I	0	0	1	0	1	0	n.a	n.a	1		

*S* Susceptible, *R* Resistance, *I* Inconclusive, *n.a.* Not available

Total number of the conducted faecal egg count reductions across all trials: *n* = 16

Resistance and low-resistance assignments were considered to fall within the resistance category, as per the revised 2023 WAAVP guideline [40]

One reason for disagreement between statistical methods could be a study design with too low power. We therefore conducted a power analysis using simulated data. Since the lowest mean EPG before treatment in the present study was slightly above 1000 and the multiplication factor was 5 (cattle) and 10 (sheep and goats) for the Mini-FLOTAC analysis, raw mean egg counts of 1000 were used to model negative binomial distributed pre-treatment egg count values. The number of animals was varied between 5 and 50, and the overdispersion parameter was varied between 0.1 and 2. For the susceptibility test, true efficacies between 0.99 and 1 were assumed, while for the resistance test, true efficacies between 0.8 and 0.99, with the majority between 0.95 and 0.99, were assumed. The results are shown for all three test methods in Additional file 9: Figs S1–S6. In addition, the overdispersion of the FEC data before treatment in the present study (Additional file 10: Table S4). The data clearly show that the power to detect resistance with the observed k values in the range 1.0–2.4 was above 80.0% if at least 10 animals were used and the true efficacy was 97.0% or lower. The high k values, i.e. low overdispersion, observed in the present study also suggest that the delta method, which assumes a Poisson distribution (k = 1), is a fairly good approximation.

Finally, the two eggCounts methods were compared in terms of how well they fitted the actual data. For this purpose, k-fold cross-validation was performed. The results are provided in Additional file 11, Table S5. For all FECRTs conducted in the present study, the model with individual efficacy showed a significantly (*P* < *0.05*) better fit to the data than the corresponding model with common efficacy.

## Discussion

To the authors’ knowledge, this is the first study in Sudan, and if not the first, one of the few in Africa, to comprehensively evaluate the efficacy of levamisole across three ruminant species (cattle, sheep and goats), combining natural and experimental efficacy assessments, including monitoring for toxicity. The ruminants studied exhibited mixed GIN infections, including different strongyles and *S. papillosus*. Strongyle cultures identified three genera/groups: *Haemonchus*, *Trichostrongylus* and *Oesophagostomum*/*Chabertia* (Chabertiidae). Among these, *Haemonchus* spp. dominated, with relative abundances of 71.0% in cattle, 74.0% in sheep and ranging from 72.0% to 89.0% in goats among the four study regions.

The revised 2023 WAAVP guideline [40] recommends the criteria outlined by Denwood et al. [44], the use of a non-parametric model with common efficacy, for data analysis. The default delta method in bayescount, as implemented in the fecrt.com web page, does not take into account the additional precision that comes with low multiplication factors at all. Therefore, the parametric models for common efficacy and individual efficacy provided by the eggCounts package were also employed. These models were used to understand the effects of the statistical models to interpret the results of the FECRT and to highlight any differences that may have arisen when applying the three models, thereby providing more robust conclusions. This may also contribute to the improvement and/or development of future statistical models. In cases where discrepancies occurred between the models, the final assignment for susceptibility or resistance followed the recommendations of the revised 2023 WAAVP guideline (bayescount) [40]. The FECR data showed substantial agreement with both the parametric model with individual efficacy and the non-parametric model with common efficacy (Cohen’s κ coefficient: 0.758; 95% CI: 0.447–1.069), as well as between the parametric and non-parametric models for common efficacy (Cohen’s κ coefficient: 0.765; 95% CI: 0.484–1.045) [45]. Moderate agreement was found between the parametric models with common and individual efficacy (Cohen’s κ coefficient: 0.529; 95% CI: 0.196–0.863). One reason for the three methods not being in almost perfect agreement can be easily explained by the low level of resistance. In low-resistance situations, the 90% CLs are slightly below the cut-offs of 99% and 95%, and even small changes in the CIs can lead to an entirely different interpretation of the data. In sheep in Nyala Domaia, the 90% CI for the parametric model with individual efficacy for strongyle nematodes was 95.7–99.7%, while a 90% CI of 91.8–93.4% was calculated for the eggCount parametric model with common efficacy. The non-parametric model with common efficacy had a 90% CI of 88.0–96.2%. Strongyle efficacy in goats in Tulus had 90% CIs close to the cut-off values of 99% and 95% including 96.6–99.1% for the parametric model with individual efficacy, 95.8–96.5% for the parametric model with common efficacy, and 94.4–97.7% for the non-parametric model with common efficacy, respectively. The difference between these three models and the effect of low resistance on the analysis have recently been highlighted [33, 46].

A power analysis using simulated data was performed to exclude that the relatively low agreement between methods is due to too low power of the analyses. The fact that the calculated overdispersion parameters were low and the variances in the datasets from the present study were thus low, the power analysis suggests that the disagreement of different statistical methods was not due to low power of the study design/data distribution. These data suggest that the common efficacy model, with a low number of model parameters, has by far the highest power, and both eggCounts models never fail, i.e. they are always able to calculate a 90% CI. In contrast, the delta method failed frequently with increasing overdispersion (lower k values). This does not lead to failure of bayescount in total, since the online version does switch to BNB methods automatically and decides about the resistance/susceptibility status of the strongyle community without providing any confidence intervals. These methods were not included in the current method comparisons.

In addition, comparisons of both parametric models were based on log-likelihoods of the fitted models. In all cases, the model with individual efficacy was significantly better in the k-fold cross-validation analyses. This occurred despite of the fact that the individual efficacy model contains a much higher number of parameters (e.g. individual efficacy for each animal) and this is inherently penalised by k-fold cross-validation. One reason for this observation might be that, also in the resistant communities, several animals showed high FECRi values. Particularly for goats, the dispersion of the FECRi values was very high, and this is where the majority of disagreements between methods were observed. Applying k-fold cross-validation can in the future be used to compare different eggCounts models to apply in the end only models with the minimum number of parameters to achieve a good fit. The power analysis shows that increasing the number of parameters results in a substantial loss in power.

Data on parasites of cattle from the present study from Bulbul showed that these parasite communities were susceptible to levamisole, with all three statistical models, for both identified GINs: strongyles (FECR of parametric models: 98.8–100%; 90% CI of parametric and non-parametric models: 97.9–100%) and *S. papillosus* (FECR: 99.8–99.9%; 90% CI: 98.3–100%). These efficacies are higher than those reported in a previous study on calves naturally infected with levamisole-susceptible GINs in Argentina, where SC treatment (8 mg/kg bw) achieved FECRs of 93.0–99.0% (95% CI: 80.0–99.0%) [47]. A 100% efficacy for levamisole has been reported in cattle naturally infected with GINs in Nigeria [48]. Similarly, a study in Kenya observed 95.0% efficacy at a 7.5 mg/kg bw dose [28]. Despite decades of global availability, levamisole use has been less frequently used than other anthelmintics due to its narrow therapeutic index [16, 17], presumably resulting in lower selection pressure on cattle GINs and less resistance problems in GINs of cattle in many regions [7].

Levamisole resistance of GINs of small ruminants has been increasingly reported worldwide. In the present study, reduced efficacy of levamisole in strongyle nematodes in sheep and goats has been observed. In sheep from Nyala Domaia, both parametric and non-parametric models with common efficacy for all animals indicated levamisole resistance (FECR (common efficacy): 92.6%; 90% CI range: 88.8–96.2%) according to the revised 2023 WAAVP guideline [40]. However, a parametric model with individual efficacy suggested susceptibility (FECR: 98.2%; 90% CI: 95.7–99.7%). Despite differences among the models, levamisole resistance in strongyles in sheep in Nyala Domaia is not surprising. In Khartoum, Sudan, Alnaeim et al. [49] reported reduced levamisole efficacy in sheep (FECR: 56.4–91.0%) after a 7.5 mg/kg bw dose. However, their conclusion on resistance development was limited by a small sample size (8 sheep) and prior treatments with albendazole and ivermectin. In Kenya, levamisole resistance was observed on 7 of 25 sheep farms (FECR: 62.0–93.0%) [50]. Lower efficacy (FECR: 53–81% (levamisole dose: 7.5 mg/kg bw)) was reported in Trinidad [51], while in South Africa, FECR ranged from 70.0–94.0% after a 5 mg/kg bw dose [22]. Reduced levamisole efficacy in sheep was also reported from Australia [19], Asia [24, 26], Europe [52–54], the United States [23] and South America [20, 21].

Levamisole efficacy against GINs in goats was studied in four regions of South Darfur using two doses. At 12 mg/kg bw, tested only in Nyala Domaia, reduced efficacy was observed, with a FECR of 93.3–95.3% (90% CI: 88.3–97.4%) to the three statistical models used. However, due to toxicity (mild toxicity [39]) in 40.0% (4/10) of the tested goats, the dose was reduced to 10 mg/kg bw in all further studies. This dose, tested in Bulbul, Kass, Nyala Domaia and Tulus, showed reduced efficacy against strongyles in the first three regions (FECR range: 85.5–95.0%; 90% CI: 77.7–97.5%). In Tulus, while parametric (FECR: 96.2%; 90% CI: 95.8–96.5%) and non-parametric models (90% CI: 94.4–97.7%) indicated resistance/low resistance, a parametric model with individual efficacy for each animal suggested susceptibility (FECR: 98.0%; 90% CI: 96.6–99.1%) [40]. Despite this discrepancy, findings confirm levamisole-resistant strongyle nematodes in the four regions, even at the recommended 1.5-fold sheep dose of 12 mg/kg bw or the adjusted 10 mg/kg bw dose. Experimental infection of goats with a *H. contortus* isolate from Um Dafuq, a fifth region with suspected levamisole-susceptible parasites (based on lower levamisole use as reported by local veterinary drug distributors), confirmed that *H. contortus* in the local goat breed are principally susceptible to levamisole at 8 mg/kg bw. The FECR ranged from 98.4–100% (parametric models) with a lower 90% CI of 95.0–99.3% and an upper 90% CI of 98.7–100% across all models. These results confirm that reduced efficacy of strongyle nematodes in the other regions was due to resistance evolution and not an insufficient dose. The 93.3% efficacy (parametric model with common efficacy) observed for goats in Nyala Domaia with a dosage of 12 mg levamisole/kg bw SC is still higher than the 80.0–83.0% efficacy reported for goats in Mexico with the same dose and application route [55]. Even lower efficacies (FECR range: 49.0–56.0%) have been observed after oral levamisole administration at 12 mg/kg bw in India [56], while higher efficacy (FECR range: 95.0–100%) has been reported after oral administration at the same dose in Poland [57]. The efficacy of the 10 mg levamisole/kg bw against strongyles in South Darfur (85.5–96.2% FECR range for the parametric model with common efficacy) compares to 96.0% efficacy in goats at a dose of 8 mg/kg bw SC in Nigeria [27], exceeds the 29.0–97.0% range reported at 10.5 mg/kg bw in Uganda [11] and is higher than the 79.4% observed at 7.5 mg/kg bw orally in Brazil [20]. Levamisole resistance in goats is an issue that has been reported globally, including reports from Australia [19], Asia [24, 58], Europe [53, 59], the United States [23] and South America [20, 55].

In the present study, *S. papillosus* eggs were not detected under the microscope in sheep but were observed in three regions in goats, including Bulbul, Kass and Nyala Domaia, before treatment. A 10 mg/kg bw dose of levamisole successfully removed *S. papillosus* eggs in the faeces of treated goats from Kass and Nyala Domaia. However, results from Bulbul were considered inconclusive based on the parametric model with individual efficacy and the non-parametric model with common efficacy to all animals. The parametric model with common efficacy to all animals classified *S. papillosus* in goats from Bulbul as levamisole-resistant (FECR: 91.3%; 90% CI: 82.5–96.2%) [40]. The high efficacy of levamisole against *S. papillosus* in goats from Kass and Nyala Domaia aligns with many previous reports indicating full efficacy [10, 11, 20]. However, none of the data available highlighted levamisole resistance in this parasite.

Signs of levamisole toxicity were observed in sheep and goats but not in cattle. In sheep, only one animal displayed symptoms of neurological toxicity at the recommended dose, whereas more goats exhibited toxic effects. At a levamisole dose of 12 mg/kg bw, 1.5-times the recommended sheep dose and the recommended dose for goats according to Myers et al. [18], 40.0% of the goats observed mild cholinergic toxicity, including hyperactivity, ataxia and muscle tremors. The adjusted 10 mg levamisole/kg bw dose resulted in milder symptoms in 3 out of 107 goats, which disappeared within 30 min. At a dose of 8 mg/kg bw, administered SC to goats infected experimentally with *H. contortus*, no toxicity was observed. The severity of levamisole toxicity in sheep at the recommended dose and in goats using 10 and 12 mg/kg bw doses was assigned as mild, as explained previously [39]. Levamisole has been identified as having a narrow therapeutic index, with toxic levels potentially reached at as little as twice the recommended dose. This is due to its nicotine-like structure and effects [16, 17]. Several factors may have influenced levamisole toxicity, including animal species, age, health, body condition and the route of administration [17]. An additional factor suggested by the results of the present study is the animal breed. Desert sheep and goats, the local breeds in South Darfur, exhibited signs of mild toxicity [39], particularly at the 12 mg levamisole/kg bw dose administered subcutaneously to goats. However, none of many of the previous studies using other goat and sheep breeds highlighted toxicity at this dose (12 mg/kg) [55–57]. However, most of these studies involved oral administration, and it is therefore unclear if differences in toxicity are due to animal breed or application route [17]. Malla et al. [60] evaluated the pharmacokinetic properties of levamisole in goats following SC injection at a single dose of 9 mg/kg bw and oral administration at a single dose of 12 mg/kg bw. However, the SC route resulted in a lower maximum plasma concentration (C_max_: 468.57 ± 151.12 ng/mL) compared with the oral route (573.21 ± 149.01 ng/mL) and the drug was eliminated more rapidly.

Although levamisole toxicity in goats in the present study at a dose of 12 mg/kg bw was classified as mild in 4/10 animals [39], continuing the study at this dose for the remaining 107 goats could have led to farmers’ disagreement and may raise animal welfare concerns. Moreover, veterinarians in South Darfur commonly estimate animal body weight visually when calculating drug dosages, and farmers often administer a constant dose to young and adult animals [3]. These practices increase the risk of dosing errors and, consequently, the likelihood of levamisole toxicity and a higher severity of clinical symptoms. Therefore, the dose was reduced from 12 mg/kg bw to 10 mg/kg bw.

Estimation of animal body weight using body measurements is a common practice in farm animals, particularly for determining drug dosages [61]. In Sudan, this method has been evaluated and recommended for estimating live body weight in local sheep and goat breeds [62, 63]. The expected error margin is below 10%, which falls within the ± 20% body weight error range considered acceptable for animal dosing [61, 64].

Host species is recognised as a factor influencing anthelmintic efficacy [40], due to differences in levamisole pharmacokinetics between cattle, sheep and goats [18, 47]. Additional factors include the region and the GIN species. Previous studies in South Darfur have shown that the predominance of different *Haemonchus* species in cattle (*H. placei*) and small ruminants (*H. contortus*) [3, 6] as well as variations in pasture contamination with resistant nematode communities within regions of the same climate zone [3], play an important role in the efficacy of anthelmintics. The present study found that host species was a factor significantly (*P* < *0.01*) associated with resistance to levamisole. Bivariable logistic regression analysis in Bulbul showed higher odds for individual animals to show a FECRi below 99% for cattle (odds ratio: 21.714; 95% CI: 6.580–85.572; *P* < *0.001*) compared to goats. However, analysing FECR data for cattle, sheep and goats across all four regions is not fully representative, since all three host species were not studied in each region, it nevertheless provided valuable insights into the resistance status of GIN communities in the examined animals. The GLMM analysis revealed significant differences between cattle and sheep (odds ratio: 5.429; 95% CI: 1.700–17.337; *P* = *0.004*) and between cattle and goats (odds ratio: 13.818; 95% CI: 5.612–34.025; *P* < *0.01*), indicating that levamisole resistance is significantly associated with strongyle nematodes (*H. contortus*) of small ruminants. Similarly, the beta-regression analysis for FECRi showed that the reductions were lower in goats than in cattle and sheep, which displayed an intermediate response. Unexpectedly, there was also a poorer response in female animals compared to males.

Faecal cultures from sheep and goats after levamisole treatments at different doses showed that only *Haemonchus* spp. survived the treatment. This finding aligns with numerous previous studies involving levamisole treatments [11, 19, 65]. Presence of *Haemonchus* spp., particularly *H. contortus*, as the surviving nematode following anthelmintic treatment has also been observed in South Darfur in cattle and goats treated with albendazole [3, 6], as well as in sheep and goats treated with ivermectin [33]. Nonetheless, microscopic differentiation of *Haemonchus* spp. L3 alone does not rule out the possibility of other strongyle genera/groups, such as *Trichostrongylus* spp. and/or *Oesophagostomum*/*Chabertia*, being present since only 100 L3 were counted, and the frequencies of other genera may be very low. This is supported by our previous study in cattle in South Darfur, Sudan. While microscopic examination of L3 pooled samples after albendazole treatment only identified *Haemonchus* spp., genus-specific PCRs detected more genera in some samples in addition to *Haemonchus* spp., including *Trichostrongylus* spp. (8 out of 13 samples) and *Cooperia* spp. (7 out of 13 samples) [6]. An additional limitation of coproculture is that optimal culture conditions might differ between strongyle species, and thus, the environmental parameters, such as temperature and humidity, might have an effect on the observed species distribution.

The findings of the present study, along with our previous reports on benzimidazole and ivermectin resistance in sheep and goats in South Darfur [3, 33], highlight the emerging problem of anthelmintic resistance, particularly in parasites of small ruminants. In general, this phenomenon significantly impacts parasite control due to persisting infections, reduced animal production, increasing the costs of treatment, and, most critically, threatening food security in the region [9].

Advanced molecular biology techniques, such as deep amplicon sequencing to identify strongyle nematodes before and after treatment, as recently applied to goats in Mozambique [66], could play an important role in understanding the composition of strongyle nematodes before and after treatment in Africa. Such analyses can be based on archived material from the present study. In addition, deep amplicon sequencing methods to quantify the presence of a levamisole resistance marker in *Haemonchus* spp. have been described [19] and should be applied in the future. Moreover, the collection of data on animal husbandry practices and anthelmintic treatments will enable a better understanding of the impact of grazing and treatment practices employed by farmers in the study regions on the development of levamisole resistance.

## Conclusions

This study has demonstrated the presence of levamisole resistance in *Haemonchus* spp. in sheep and goats in South Darfur (Sudan). Next steps in the region should include evaluation of the efficacy of anthelmintic combinations, improvement of farm management practices, implementation of targeted selective treatments and understanding of the molecular mechanisms causing multi-drug-resistance of *Haemonchus* spp.

## Supplementary Information

Supplementary Material 1. R code and Slurm scripts used for running power analyses on a high-performance computer (HPC) for sensitivity and resistance tests across three statistical methods: the parametric model with individual efficacy for each animal (eggCounts), the parametric model with common efficacy for all animals (eggCounts) and the non-parametric model with common efficacy for all animals (default in bayescount).

Supplementary Material 2. R code used for the calculation of the fit of the models with common and individual efficacy calculated with eggCounts.

Supplementary Material 3. R code used for the calculation of levamisole efficacy when evaluated separately for parasite communities across host species, regions and doses.

Supplementary Material 4. R code used for the calculation of beta regression analysis concerning levamisole efficacy when evaluated separately for parasite communities across host species, regions and doses.

Supplementary Material 5: Table S1. Raw faecal egg count data were collected from cattle, sheep and goats naturally infected with gastrointestinal strongyle nematodes and from goats experimentally infected with *Haemonchus contortus*.

Supplementary Material 6: Table S2. Raw faecal egg count data were collected from cattle and goats naturally infected with *Strongyloides papillosus*.

Supplementary Material 7: Video S1. Clinical symptoms in a desert goat in Nyala Domaia, South Darfur, Sudan, following subcutaneous administration of levamisole at a dose of 12 mg/kg body weight.

Supplementary Material 8: Table S3. Variables with influence on individual faecal egg count reduction (FECRi, scale 0 -1) are identified by beta-regression.

Supplementary Material 9: Figs S1–S6. Power calculations for the resistance and susceptibility tests for bayescout delta method and eggCounts with and without individual efficacy.

Supplementary Material 10: Table S4. Faecal egg count reduction (FECR) with 90% confidence intervals (CIs), including estimated over-dispersion (k) for strongyles, before and after treatment with levamisole, subcutaneous injection, in cattle, sheep and goats, South Darfur, Sudan.

Supplementary Material 11: Table S5. Comparison of eggCounts models with common and individual efficacy for all faecal egg count reduction tests (FECRTs).

## Data Availability

The relevant information, including raw data, has been included in the manuscript.

## Abbreviations

AIC: Akaike Information Criterion
CI: Confidence interval
CL: Confidence limit
LCL: Lower confidence limit
UCL: Upper confidence limit
EPG: Eggs per gram
FEC: Faecal egg count
FECR: Faecal egg count reduction
FECRi: Individual faecal egg count reduction
FECRT: Faecal egg count reduction test
GINs: Gastrointestinal nematodes
HPC: High-performance computer
L3: Third-stage larvae
MCMC: Markov Chain Monte Carlo
PSIS-LOO: Pareto-smoothed importance sampling leave-one-out
SC: Subcutaneously
SD: Standard deviation
WAAVP: World Association for the Advancement of Veterinary Parasitology
WAIC: Watanabe–Akaike Information Criterion
