# Supplementary material for: Variability in the anthelmintic efficacy of levamisole against gastrointestinal nematodes of cattle, sheep and goats in South Darfur, Sudan
Source: BMC Vet Res. 2026 Feb 11;22:128. doi: 10.1186/s12917-026-05320-2 (PMC12930928; doi:10.1186/s12917-026-05320-2)
Supplement: Supplementary file 8 — Supplementary Material 8: Table S3. Variables with influence on individual faecal egg count reduction (FECRi, scale 0 -1) are identified by beta-regression. [file 12917_2026_5320_MOESM8_ESM.pdf]

Variability in the anthelmintic efficacy of levamisole against gastrointestinal nematodes of cattle, sheep and goats in South Darfur, Sudan

Khalid M. Mohammedsalih[1,2,3,4], Abdoelnaim I. Y. Ibrahim[4], Fathel-Rahman Juma[3,4], Abdalhakaim A. H. Abdalmalaik[4], Ahmed Bashar[4], Georg von Samson-Himmelstjerna[1,2], Jürgen Krücken[1,2]

---

[1]Institute for Parasitology and Tropical Veterinary Medicine, Freie Universität Berlin, Robert-von-Ostertag-Str. 7, 14163 Berlin, Germany

[2]Veterinary Centre for Resistance Research, Freie Universität Berlin, 14163 Berlin, Germany

[3]Central Research Laboratory of Darfur Universities, Mousseh district, 63311 Nyala, Sudan

[4]Faculty of Veterinary Science, University of Nyala, Mousseh district, 63311 Nyala, Sudan

Corresponding author: juergen.kruecken@fu-berlin.de

Additional file 8. Variables with influence on individual faecal egg count reduction (FECRi, scale 0 -1) are identified by beta-regression.

Table S3

Variables with influence on individual faecal egg count reduction (FECRi, scale 0 -1) are identified by beta-regression.

		**Logit scale**				**FECRi scale (0-1)**		
	Variable	Estimate	Standard error	Z value	*P* value	Estimate	Lower confidence limit	Upper confidence limit
Conditional means	Intercept	3.5	0.25	14.45	<0.001	0.97	0.96	0.98
	Cattle (reference)	1						
	Goats	-1.37	0.26	-5.26	<0.001	0.20	0.13	0.30
	Sheep	0.99	0.37	2.69	0.007	0.27	0.15	0.43
	Female (reference)	1						
	Male	0.75	0.25	2.98	0.003	0.68	0.56	0.78
Dispersion		Precision φ[a]	Standard error	Z value	*P* value			
	Intercept (cattle, female)	2.52	0.30	8.52	<0.001	NA		
	Goats	-0.90	0.32	-2.82	0.005	NA		
	Sheep	0.70	0.45	1.57	0.117	NA		
	Male	1.39	0.35	4.01	<0.001	NA		

[a]φ increases with lower dispersion.
