# Supplementary material for: Variability in the anthelmintic efficacy of levamisole against gastrointestinal nematodes of cattle, sheep and goats in South Darfur, Sudan
Source: BMC Vet Res. 2026 Feb 11;22:128. doi: 10.1186/s12917-026-05320-2 (PMC12930928; doi:10.1186/s12917-026-05320-2)
Supplement: Supplementary file 9 — Supplementary Material 9: Figs S1–S6. Power calculations for the resistance and susceptibility tests for bayescout delta method and eggCounts with and without individual efficacy. [file 12917_2026_5320_MOESM9_ESM.pdf]

Variability in the anthelmintic efficacy of levamisole against gastrointestinal nematodes of cattle, sheep and goats in South Darfur, Sudan

Khalid M. Mohammedsalih[1,2,3,4], Abdoelnaim I. Y. Ibrahim[4], Fathel-Rahman Juma[3,4], Abdalhakaim A. H. Abdalmalaik[4], Ahmed Bashar[4], Georg von Samson-Himmelstjerna[1,2], Jürgen Krücken[1,2]

[1]Institute for Parasitology and Tropical Veterinary Medicine, Freie Universität Berlin, Robert-von-Ostertag-Str. 7, 14163 Berlin, Germany

[2]Veterinary Centre for Resistance Research, Freie Universität Berlin, 14163 Berlin, Germany

[3]Central Research Laboratory of Darfur Universities, Mousseh district, 63311 Nyala, Sudan

[4]Faculty of Veterinary Science, University of Nyala, Mousseh district, 63311 Nyala, Sudan

Corresponding author: juergen.kruecken@fu-berlin.de

Additional file 9. Power calculations for the resistance and susceptibility tests for bayescout delta method and eggCounts with and without individual efficacy.

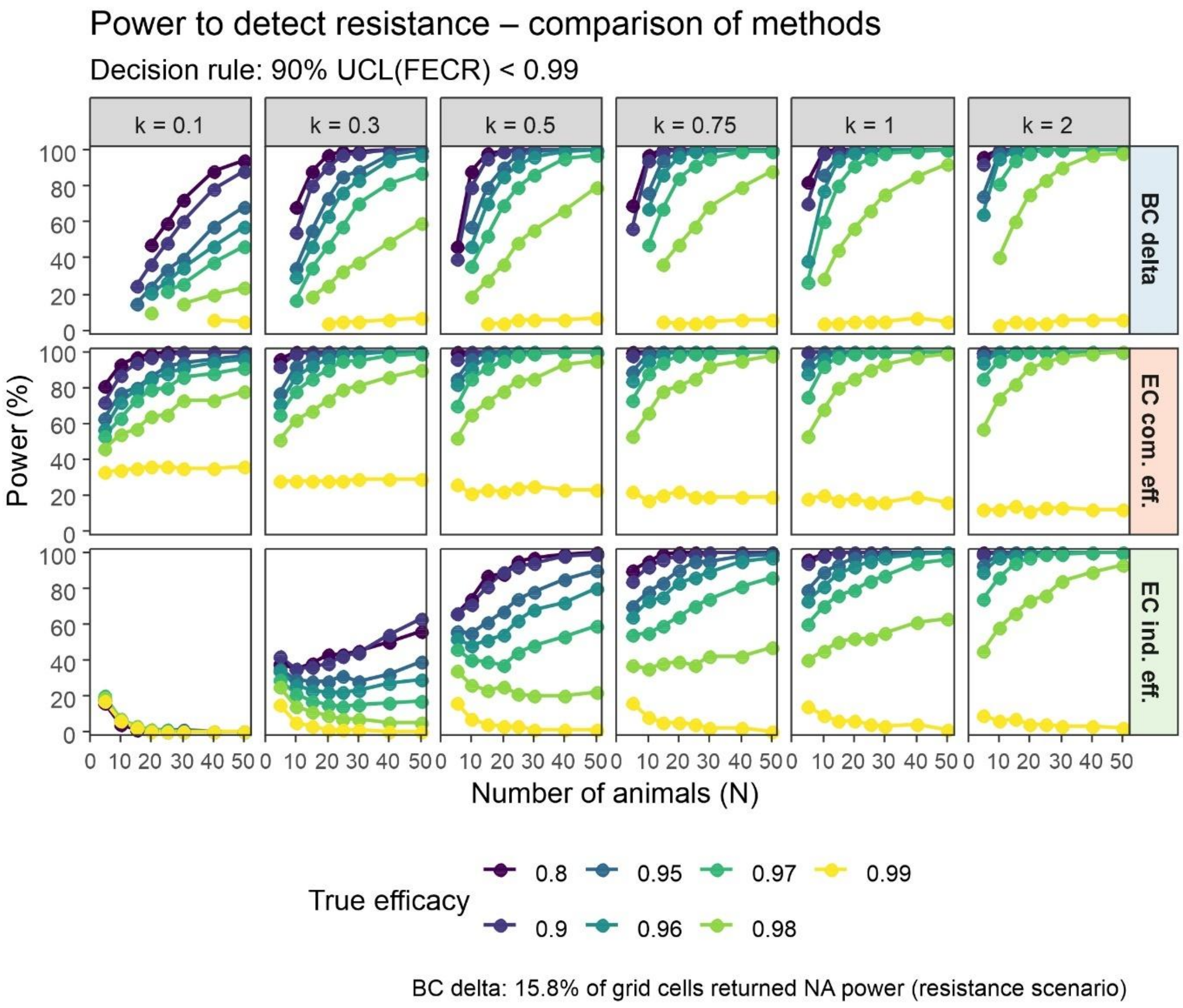

Fig. S1. Power calculations for the resistance test. Power was calculated as the percentage of times analysis of 1000 random, negative binomial distributions before (mean raw egg counts = 100) and after treatment were detected by the bayescount (BC) delta, the eggCounts (EC) with common (com) efficacy and individual (ind.) efficacy were detected as resistant. Overdispersion parameter k, number of animals N and true efficacies were varied. In a considerable number of cases (15.8%), the bayescount delta method failed to calculate any 90% confidence in all of the 1000 replicates and NA was produced as result. LCL, lower confidence limit; UCL, upper confidence limit.

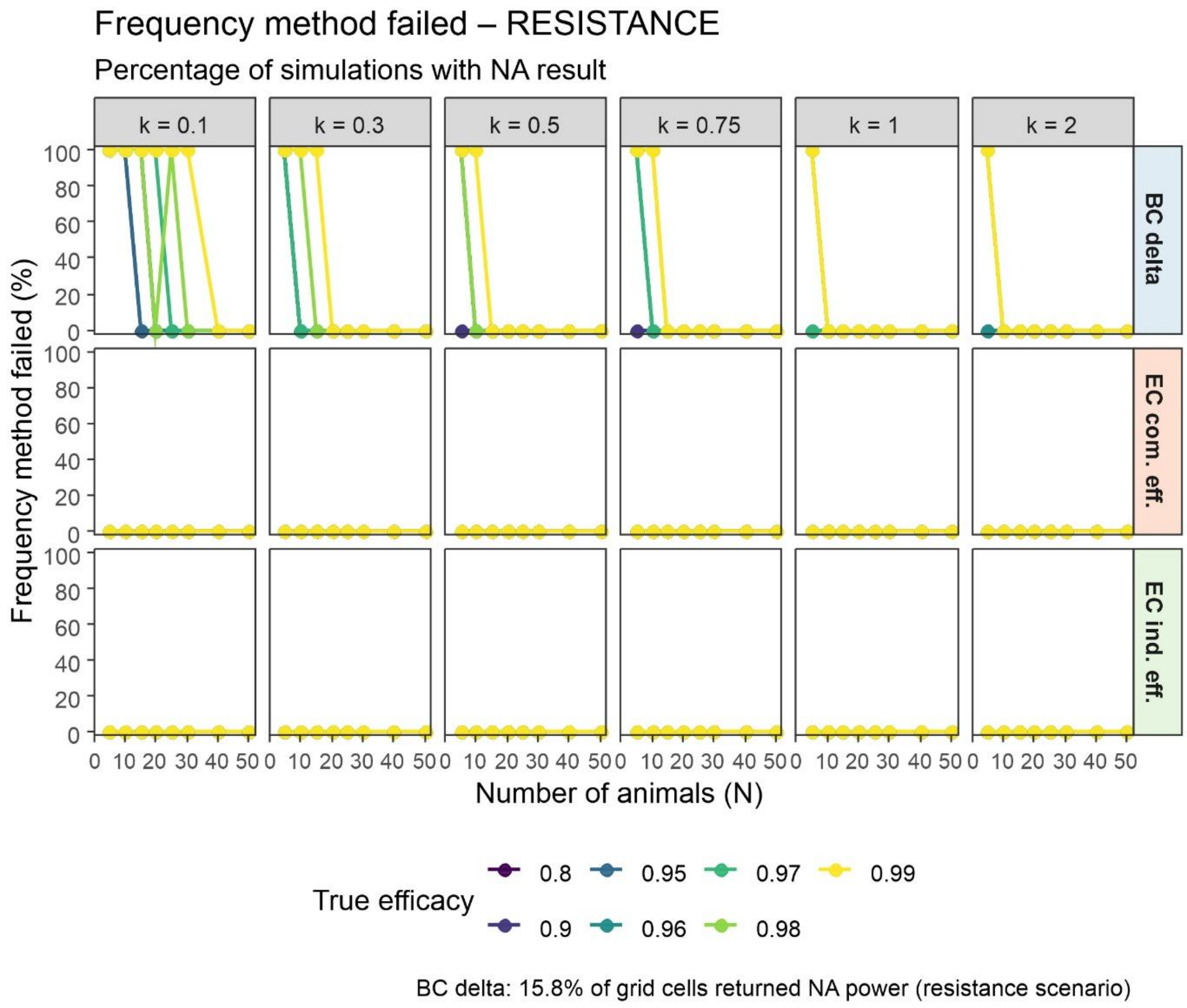

Fig. S2. Frequency of calculation failures for the resistance test. The frequency was calculated as the percentage of times that the analysis of all 1000 random, negative binomial distributions before (mean raw egg counts = 100) and after treatment did not provide any 90% confidence interval was obtained. The bayescount (BC) delta, the eggCounts (EC) with common (com) efficacy and individual (ind.) efficacy were used. Overdispersion parameter k, number of animals N and true efficacies were varied.

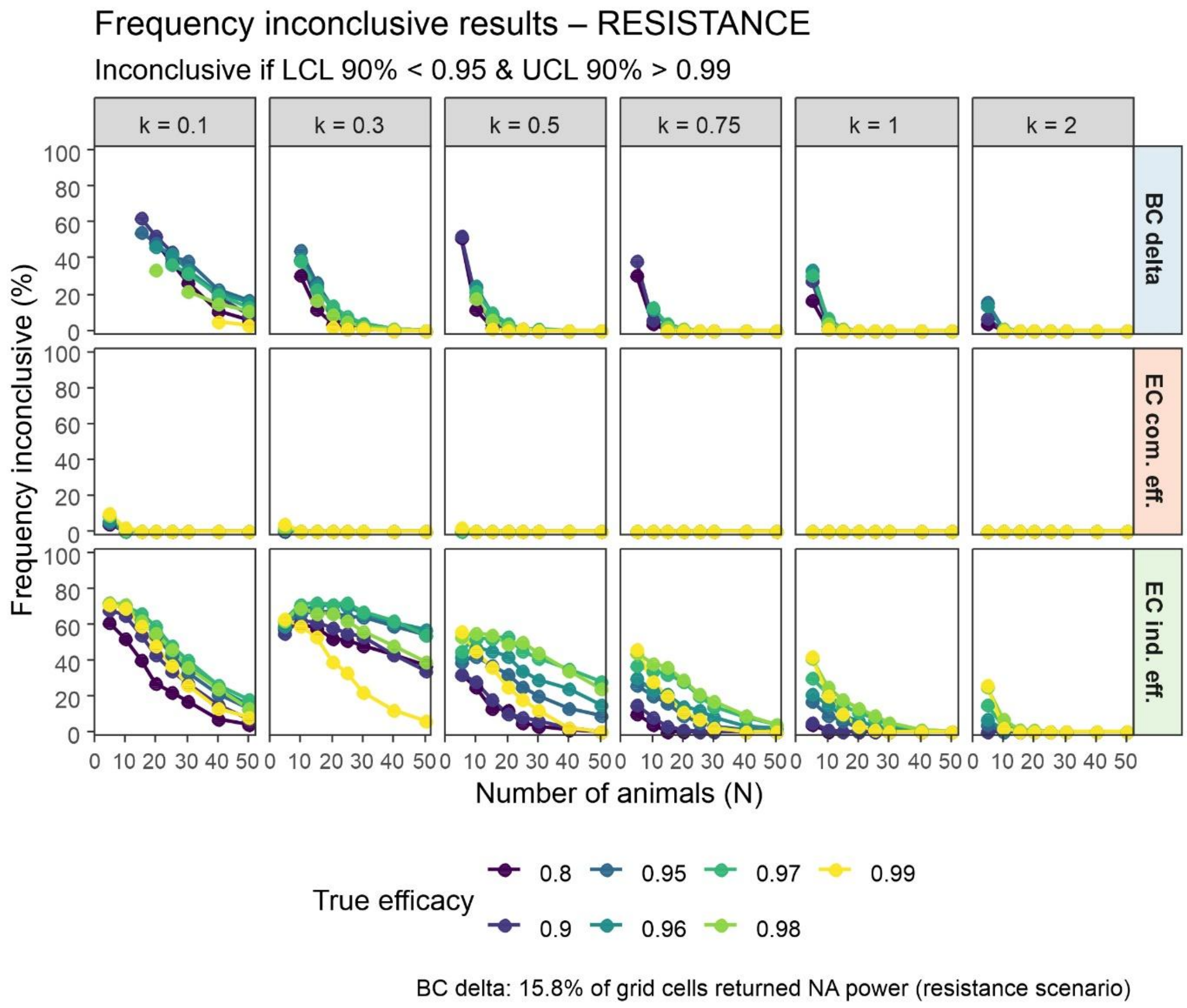

Fig. S3. Frequency of inconclusive results for the resistance test. The frequency was calculated as the percentage of times analysis of 1000 random, negative binomial distributions before (mean raw egg counts = 100) and after treatment led to inconclusive results (i.e. lower 90% LCL <95% and 90% UCL >99%), were obtained. The bayescount (BC) delta, the eggCounts (EC) with common (com) efficacy and individual (ind.) efficacy were used. Overdispersion parameter k, number of animals N and true efficacies were varied. LCL, lower confidence limit; UCL, upper confidence limit.

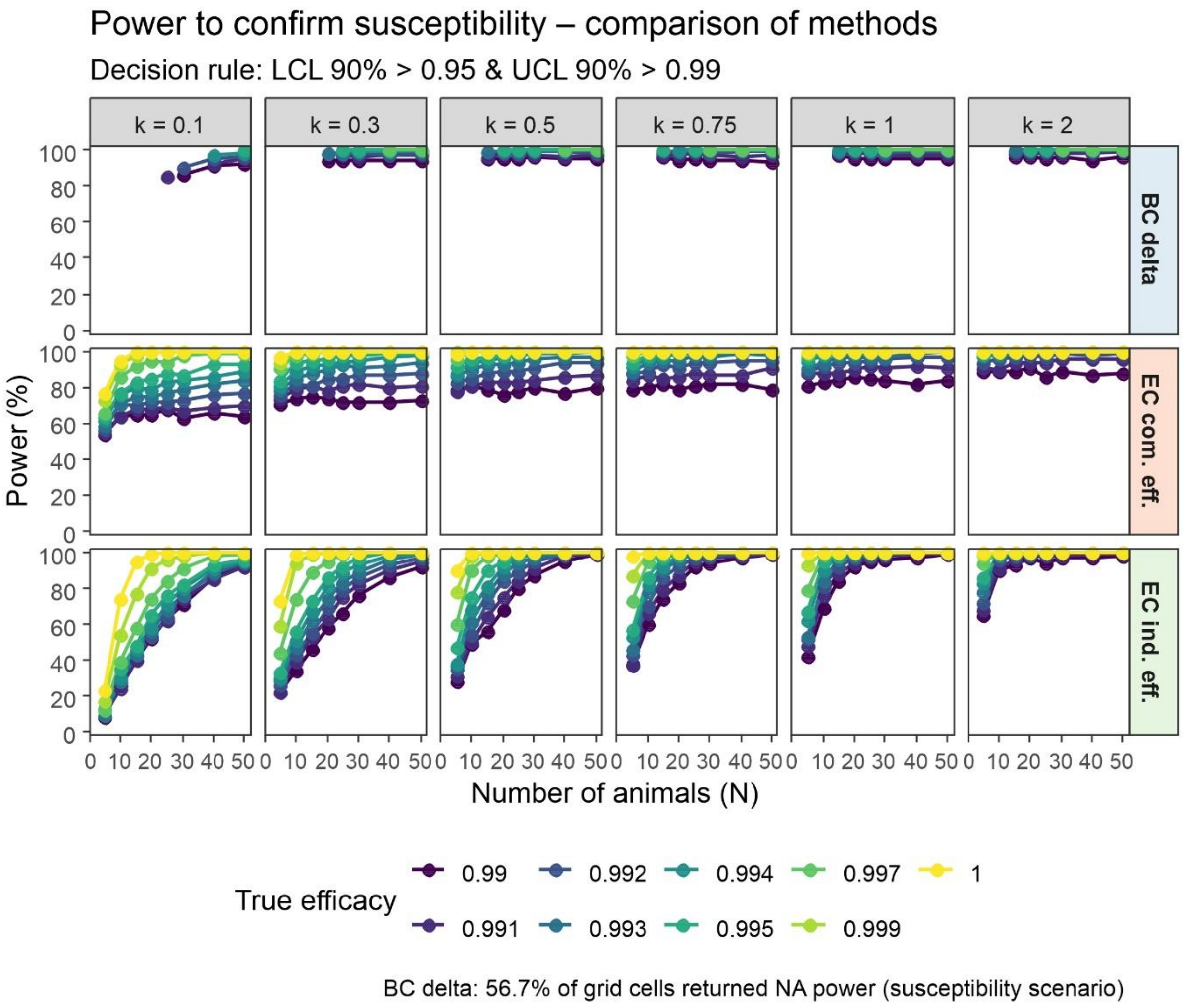

Fig. S4. Power calculations for the susceptibility test. Power was calculated as the percentage of times analysis of 1000 random, negative binomial distributions before (mean raw egg counts = 100) and after treatment were detected by the bayescount (BC) delta, the eggCounts (EC) with common (com) efficacy and individual (ind.) efficacy were detected as susceptible. Overdispersion parameter k, number of animals N and true efficacies were varied. In the majority of cases (56.7%), the bayescount delta method failed to calculate any 90% confidence interval in all of the 1000 replicates, and NA was produced as result. LCL, lower confidence limit; UCL, upper confidence limit.

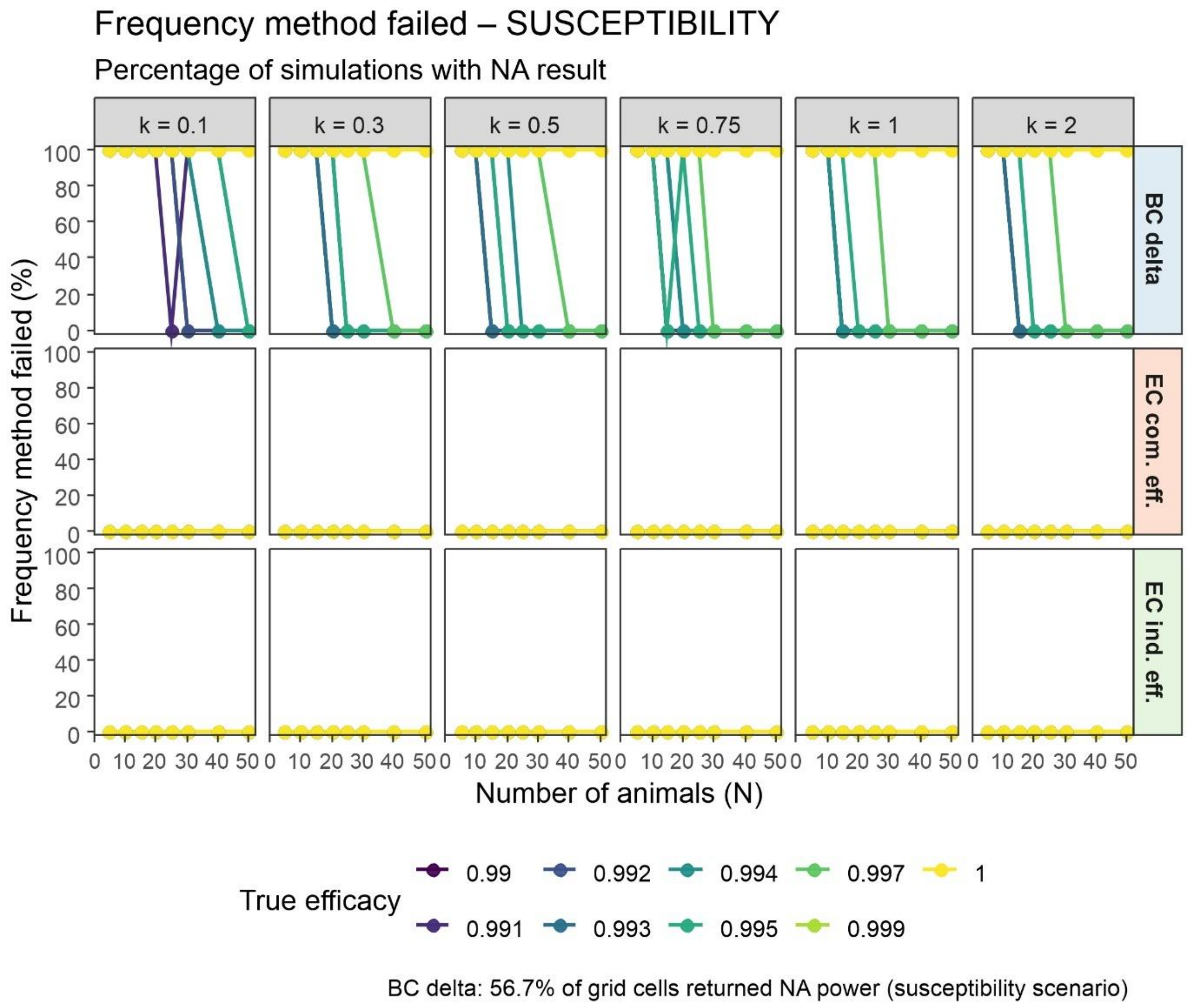

Fig. S5. Frequency of calculation failures for the susceptibility test. The frequency was calculated as the percentage of times that the analysis of all 1000 random, negative binomial distributions before (mean raw egg counts = 100) and after treatment did not provide any 90% confidence interval was obtained. The bayescount (BC) delta, the eggCounts (EC) with common (com) efficacy and individual (ind.) efficacy were used. Overdispersion parameter k, number of animals N and true efficacies were varied.

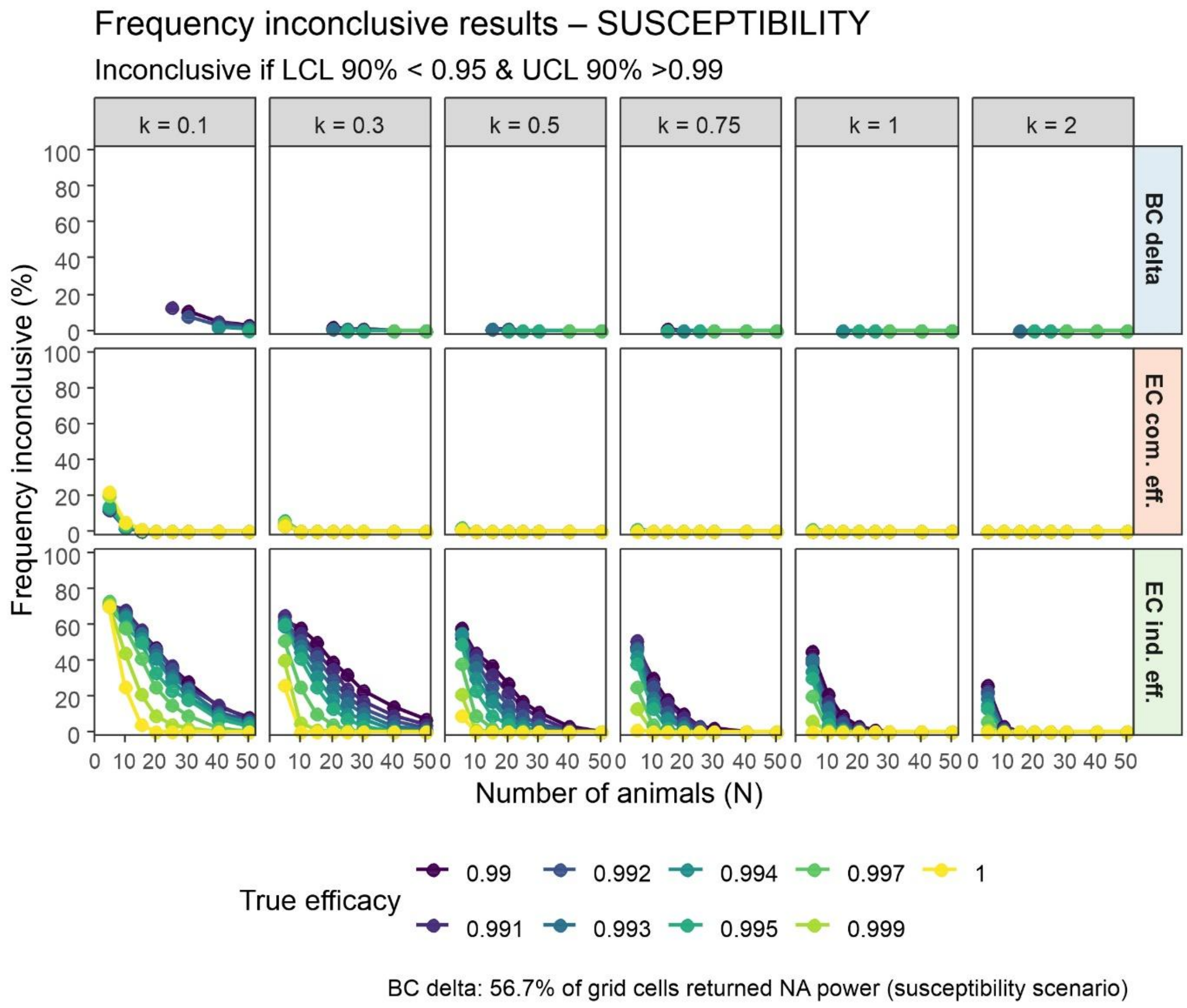

Fig. S6. Frequency of inconclusive results for the susceptibility test. The frequency was calculated as the percentage of times analysis of 1000 random, negative binomial distributions before (mean raw egg counts = 100) and after treatment led to inconclusive results, i.e. lower 90% confidence limit (LCL) <95% and 90% UCL >99%), were obtained. The bayescount (BC) delta, the eggCounts (EC) with common (com) efficacy and individual (ind.) efficacy were used. Overdispersion parameter k, number of animals N and true efficacies were varied. LCL, lower confidence limit; UCL, upper confidence limit.
