# Supplementary material for: Variability in the anthelmintic efficacy of levamisole against gastrointestinal nematodes of cattle, sheep and goats in South Darfur, Sudan
Source: BMC Vet Res. 2026 Feb 11;22:128. doi: 10.1186/s12917-026-05320-2 (PMC12930928; doi:10.1186/s12917-026-05320-2)
Supplement: Supplementary file 10 — Supplementary Material 10: Table S4. Faecal egg count reduction (FECR) with 90% confidence intervals (CIs), including estimated over-dispersion (k) for strongyles, before and after treatment with levamisole, subcutaneous injection, in cattle, sheep and goats, South Darfur, Sudan. [file 12917_2026_5320_MOESM10_ESM.pdf]

Variability in the anthelmintic efficacy of levamisole against gastrointestinal nematodes of cattle, sheep and goats in South Darfur, Sudan

Khalid M. Mohammedsalih[1,2,3,4], Abdoelnaim I. Y. Ibrahim[4], Fathel-Rahman Juma[3,4], Abdalhakaim A. H. Abdalmalaik[4], Ahmed Bashar[4], Georg von Samson-Himmelstjerna[1,2], Jürgen Krücken[1,2]

[1]Institute for Parasitology and Tropical Veterinary Medicine, Freie Universität Berlin, Robert-von-Ostertag-Str. 7, 14163 Berlin, Germany

[2]Veterinary Centre for Resistance Research, Freie Universität Berlin, 14163 Berlin, Germany

[3]Central Research Laboratory of Darfur Universities, Mousseh district, 63311 Nyala, Sudan

[4]Faculty of Veterinary Science, University of Nyala, Mousseh district, 63311 Nyala, Sudan

Corresponding author: juergen.kruecken@fu-berlin.de

Additional file 10. Faecal egg count reduction (FECR) with 90% confidence intervals (CIs), including estimated over-dispersion (k) for strongyles, before and after treatment with levamisole, subcutaneous injection, in cattle, sheep and goats, South Darfur, Sudan.

Table S4

Faecal egg count reduction (FECR) with 90% confidence intervals (CIs), including estimated over-dispersion (k) for strongyles, before and after treatment with levamisole, subcutaneous injection, in cattle, sheep and goats, South Darfur, Sudan.

Study animal	Study type	GI nematode	Dose (mg/kg bw)	No. of animals in each trial	Day of sample collection after treatment	FECR% (90% CI)			Ind- k (SD)	Com- k (SD)	k of pre-treatment data (bayescount)	k of post-treatment data (bayescount)	Estimated within-animal correlation (bayescount)
						Parametric model with individual (Ind) efficacy (eggCounts)[a]	Parametric model with common (Com) efficacy (eggCounts)[a]	Non-parametric model with common efficacy (bayescount)					
Cattle	Natural infection	Strongyles	8	45	12	100 (100-100)	98.8 (98.6-99.0)	(97.9-99.5)	1.56 (2.25)	1.568 (2.27)	1.65	0.05	0.62
		Strongyloides papillosus		5		99.8 (98.3-100)	99.9 (98.8-100)	(Uncalculatable)	n.a.	n.a.	n.a.	n.a.	n.a.
Sheep	Natural infection	Strongyles	8	22	12	98.2 (95.7-99.7)	92.6 (91.8-93.4)	(88.0-96.2)	1.66 (2.76)	1.60 (2.57)	1.89	0.18	0.85
Goats	Natural infection	Strongyles	10	25	12	94.0 (90.3-96.7)	94.2 (93.7-94.7)	(91.1-96.7)	1.09 (1.70)	1.15 (1.81)	1.15	0.65	-0.05
		S. papillosus		6		97.4 (81.8-100)	91.3 (82.5-96.2)	(72.3-99.6)	n.a.	n.a.	n.a.	n.a.	n.a.
		Strongyles	10	21		94.7 (92.2-96.6)	95.0 (94.4-95.5)	(91.7-97.5)	0.93 (1.55)	0.96 (1.59)	1.02	0.68	0.53
		S. papillosus		9		99.9 (99.2-100)	100 (99.3-100)	(Uncalculatable)	n.a.	n.a.	n.a.	n.a.	n.a.
		Strongyles	12	10		95.3 (92.3-97.4)	93.3 (92.0-94.5)	(88.3-97.2)	1.79 (3.77)	1.64 (3.62)	2.54	0.46	0.82
		Strongyles	10	30		93.3 (88.0-97.1)	85.5 (84.2-86.6)	(77.7-91.7)	2.03 (3.13)	1.96 (3.06)	2.26	0.34	0.46
		S. papillosus		7		99.6 (97.7-100)	99.9 (97.8-100)	(Uncalculatable)	n.a.	n.a.	n.a.	n.a.	n.a.
		Strongyles	10	31		98.0 (96.6-99.1)	96.2 (95.8-96.5)	(94.4-97.7)	2.14 (3.31)	2.13 (3.30)	2.38	0.38	0.44
Goats	Experimental infection	*Haemonchus contortus*	8	10	5	98.9 (97.5-99.7)	99.1 (98.8-99.3)	(98.3-99.6)	0.74 (1.68)	0.83 (1.69)	1.02	0.50	0.58
					8	99.6 (98.0-100)	99.1 (98.8-99.3)	(98.1-99.8)	0.78 (1.69)	0.84 (1.70)	1.02	0.16	0.68
					10	100 (98.9-100)	99.5 (99.3-99.6)	(98.7-99.9)	0.82 (1.67)	0.80 (1.72)	1.02	0.11	0.37
					12	99.8 (97.2-100)	99.0 (98.7-99.2)	(97.8-99.7)	0.86 (1.74)	0.84 (1.72)	1.02	0.16	0.09
					14	99.2 (95.0-100)	98.4 (98.0-98.7)	(96.6-99.6)	0.73 (1.69)	0.83 (1.73)	1.02	0.21	0.17

[a]FECRs were paired calculated by comparing data post and pre-treatment without zero-inflation option.

The results are presented in the order parametric model with individual efficacies, parametric model with common efficacy and non-parametric model with common efficacy.

Abbreviations; bw, body weight; EPG, eggs per gram faeces; GI, gastrointestinal; SD, standard deviation.
