# Supplementary material for: Variability in the anthelmintic efficacy of levamisole against gastrointestinal nematodes of cattle, sheep and goats in South Darfur, Sudan
Source: BMC Vet Res. 2026 Feb 11;22:128. doi: 10.1186/s12917-026-05320-2 (PMC12930928; doi:10.1186/s12917-026-05320-2)
Supplement: Supplementary file 11 — Supplementary Material 11: Table S5. Comparison of eggCounts models with common and individual efficacy for all faecal egg count reduction tests (FECRTs). [file 12917_2026_5320_MOESM11_ESM.pdf]

Variability in the anthelmintic efficacy of levamisole against gastrointestinal nematodes of cattle, sheep and goats in South Darfur, Sudan

Khalid M. Mohammedsalih[1,2,3,4], Abdoelnaim I. Y. Ibrahim[4], Fathel-Rahman Juma[3,4], Abdalhakaim A. H. Abdalmalaik[4], Ahmed Bashar[4], Georg von Samson-Himmelstjerna[1,2], Jürgen Krücken[1,2]

[1]Institute for Parasitology and Tropical Veterinary Medicine, Freie Universität Berlin, Robert-von-Ostertag-Str. 7, 14163 Berlin, Germany

[2]Veterinary Centre for Resistance Research, Freie Universität Berlin, 14163 Berlin, Germany

[3]Central Research Laboratory of Darfur Universities, Mousseh district, 63311 Nyala, Sudan

[4]Faculty of Veterinary Science, University of Nyala, Mousseh district, 63311 Nyala, Sudan

Corresponding author: juergen.kruecken@fu-berlin.de

Additional file 11. Comparison of eggCounts models with common and individual efficacy for all faecal egg count reduction tests (FECRTs).

Table S5

Comparison of eggCounts models with common and individual efficacy for all faecal egg count reduction tests (FECRTs)

FECRT	Common efficacy $elpd_c$	Individual efficacy $elpd_i$	Δ elpd[a]	95% CI Δ elpd	Preferred model *P < 0.05*
Cattle Bulbul	-485.4	-341.8	140.6	73.1 – 216.9	Individual efficacy
Sheep Domaia	-308.5	-211.2	97.3	55.2 – 148.6	Individual efficacy
Goats Bulbul	-546.1	-270.7	275.5	78.3 – 528.1	Individual efficacy
Goats Kass	-336.1	-219.2	116.9	34.3 – 227.9	Individual efficacy
Goats Domaia 10 mg/kg bw	-573.1	-289.7	283.4	206.2 – 365.7	Individual efficacy
Goats Domaia 12 mg/kg bw	-108.0	-93.5	14.4	1.5 – 24.6	Individual efficacy
Goats Tulus	-522.1	-316.9	205.2	126.1 – 276.8	Individual efficacy
Goats day 0 *vs.* day 5	-126.9	-107.4	19.5	3.4 – 34.2	Individual efficacy
Goats day 0 *vs.* day 8	-126.9	-100.9	26.0	1.2 – 51.0	Individual efficacy
Goats day 0 *vs.* day 10	-134.3	-96.4	38.0	14.9 – 61.4	Individual efficacy
Goats day 0 *vs.* day 12	-202.0	-104.0	98.0	34.5 – 161.5	Individual efficacy
Goats day 0 *vs.* day 14	-141.3	-107.8	138.7	52.9 – 216.1	Individual efficacy

[a]Δ elpd = $elpd_i$ – $elpd_c$.

Abbreviations: elpd, expected log predictive density; CI, confidence interval.
